# Homeobox protein B6 and homeobox protein B8 control immune-cancer cell interactions in pancreatic cancer

**DOI:** 10.1186/s43556-025-00292-5

**Published:** 2025-07-07

**Authors:** Ludivine Bertonnier-Brouty, Sara Bsharat, Kavya Achanta, Jonas Andersson, Thanya Pranomphon, Tania Singh, Tuomas Kaprio, Jaana Hagström, Caj Haglund, Hanna Seppänen, Rashmi B. Prasad, Isabella Artner

**Affiliations:** 1 Lund Stem Cell Center, Lund University, Lund, Sweden; 2 Lund University Diabetes Center, Lund University, Malmö, Sweden; 3 Department of Surgery, Helsinki University Hospital, Helsinki, Finland; 4 Translational Cancer Medicine Research Program, Faculty of Medicine, University of Helsinki, Helsinki, Finland; 5 iCAN, Digital Cancer Precision Medicine, University of Helsinki and HUS Helsinki University Hospital, Helsinki, Finland; 6 Department of Oral Pathology and Radiology, University of Turku, Turku, Finland; 7 Institute of Molecular Medicine, Finland (FIMM), Helsinki University, Helsinki, Finland

**Keywords:** Homeobox protein B6 (HOXB6), Homeobox protein B8 (HOXB8), Fetal pancreas, Pancreatic cancer, Pancreatic ductal adenocarcinoma, Tumor-associated-macrophages

## Abstract

Pancreatic ductal adenocarcinoma (PDAC) is a lethal cancer lacking effective drugs and therefore new treatment targets are needed. In this study, we define the role of homeobox protein B6 (HOXB6) and HOXB8 in controlling pancreatic cancer tumorigenesis and immune response. Transcriptomic analysis comparing human embryonic and PDAC tissue identified a large overlap of expression profiles suggesting a re-initiation of developmental programs in pancreatic cancer. Specifically, we identified the transcription factors HOXB6 and HOXB8 as potential regulators in PDAC. We described their functions in pancreatic cancer by performing transcriptomic and tumor tissue microarray analyses, in vitro assays in pancreatic and lung cancer cell lines and co-culture experiments with immune cells. Loss of *HOXB6* and *HOXB8* in pancreatic cancer cells inhibited cell proliferation, induced apoptosis and senescence and enhanced gemcitabine sensitivity. Moreover, reduced *HOXB6* and *HOXB8* expression in pancreatic and lung adenocarcinoma cell lines affected transcription of immune response pathways which resulted in an increased sensitivity of cancer cells to anti-tumorigenic activities of macrophages suggesting that the *HOXB6* and *HOXB8* immune regulatory function is conserved in different cancer types. Additionally, naïve M0 macrophages exposed to *HOXB8* deficient PDAC cells were unable to differentiate into tumor-associated macrophages, suggesting that *HOXB8* promotes the transition of initial anti-tumor macrophage to a tumor-promoting macrophage phenotype in pancreatic cancer. Our findings indicate that *HOXB6* and *HOXB8* play important roles in regulating cell proliferation, immune response, and treatment resistance to promote pancreatic cancer tumorigenesis and could be useful therapeutic targets.

**Supplementary Information:**

The online version contains supplementary material available at 10.1186/s43556-025-00292-5.

## Introduction

Pancreatic ductal adenocarcinoma (PDAC) is the most prevalent pancreatic cancer [1] with poor prognosis and low survival rate (< 10% 5-year survival rate) [2, 3]. PDAC incidences are rising, and the number of cases is projected to increase twofold in the next decade [4–6]. Low survival rates are exacerbated by the lack of effective treatment options. Current therapies often induce drug resistance, in part due to a dense tumor stroma, its specific immune cell microenvironment, and the presence of therapy-resistant cancer stem cells [7, 8]. Thus, it becomes a priority to investigate the genetic and biological features of PDAC to facilitate diagnosis and develop novel treatment strategies.

Previous studies have identified PDAC subtypes by analyzing gene expression patterns, these novel subtypes were associated with different survival outcomes, which ultimately affected treatment sensitivity [9]. According to these expression profiles and histopathological characteristics four PDAC subtypes were defined: squamous tumors mainly enriched for TP53/TP63/TGF-β signaling; pancreatic progenitor tumors preferentially expressing genes involved in early pancreatic development; immunogenic tumors with up-regulated expression of immune network genes; and aberrantly differentiated endocrine exocrine (ADEX) tumors which had increased expression of genes activating KRAS, as well as exocrine and endocrine differentiation. Within this classification, the squamous subtype was an independent poor diagnostic factor [9].

The identification of a pancreatic progenitor PDAC subtype suggests that PDAC development and embryonic pancreas development share molecular similarities. Developmental regulators prevent apoptosis and ensure cell proliferation of progenitor cells during development. However, when these regulators are mis-expressed in adult cells, they can alter cell plasticity, promote generation and survival of tumor cells thereby contributing to tumor formation [10]. In line with this, elevated expression of transcription factors critical for pancreas development has been identified in the PDAC progenitor subtype. For example, *Sox9* regulates pancreatic epithelial cell formation during embryogenesis [11] and is critical for maintaining ductal cell identity in the adult pancreas (reviewed in [12]). However, aberrant SOX9 expression has been detected in the majority of PDAC cases with poor prognosis and it has been shown that SOX9 expression promotes PDAC tumor formation [13]. Interestingly, Sox9 regulates the expression of several pancreatic transcription factors which were readily detected in PDAC cells (GATA4, GATA6, FOXA2, HNF1A), further supporting the notion that transcriptional networks important for pancreas development are reinitiated in PDAC. Most of our knowledge on pancreas development has been generated using mouse knock out models. However, human and mouse pancreas development differ in timing of progenitor cell differentiation and expression patterns of key transcription factors; for example, gene expression of *NR4A1*, *MAFF* and *EPAS1* is restricted to human tip (acinar) progenitor cells [14]. These species-specific differences limit the possibilities of identifying common molecular pathways between human pancreas development and tumor formation.

To identify novel signaling pathways important to PDAC development, we compared RNA sequencing data from human embryonic and adult pancreas with PDAC. This analysis uncovered a significant enrichment of *HOX* transcription factors in PDAC. *HOX* genes are critical for embryogenesis, particularly for anterior–posterior patterning, but are also instrumental in tumor formation [15]. Here, we show that the transcriptional regulators *HOXB6* and *HOXB8* are enriched in the embryonic and PDAC transcriptome with predominant expression in embryonic mesenchyme and malignant ductal cells. Loss of function experiments in pancreatic cancer cell lines demonstrated that *HOXB6* and *HOXB8* control cancer cell proliferation, viability, apoptosis, senescence, and sensitivity to gemcitabine. RNA-seq and ChIP-seq analyses showed that HOXB6 and HOXB8 directly regulate expression of genes critical for cell proliferation (HOXB6) and immune-cancer cell interaction (HOXB6 and HOXB8). Co-culture of *HOXB6* and *HOXB8* deficient PDAC cells with macrophages confirmed that *HOXB6* and *HOXB8* promoted tumorigenic properties of tumor-associated macrophages and protected PDAC cells from macrophage detection. Changes in immune evasion properties were also observed in a lung adenocarcinoma cell line upon reduction of *HOXB6* and *HOXB8* expression. Our results describe a novel function of *HOX* genes in tumor cell propagation and immune evasiveness in at least pancreatic and lung cancer cells.

## Results

### *HOXB6* and *HOXB8* are expressed in human pancreas development and pancreatic cancer

**Fig. 1:**
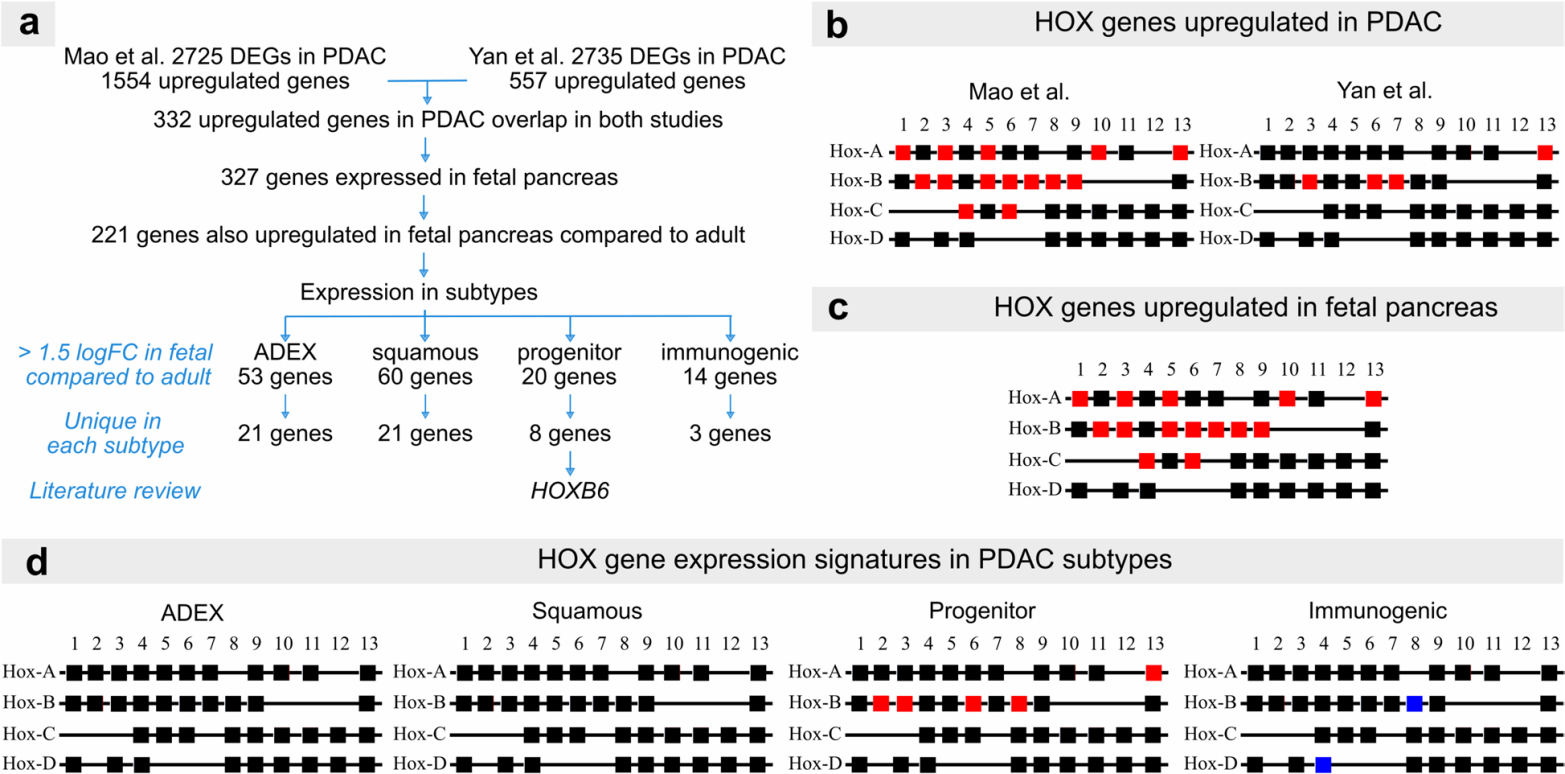
HOXB6 and HOXB8 are expressed in fetal and PDAC tissue. **a** Flow chart illustrating the workflow for identification of genes of interest. **b** Squares represent HOX genes organized by clusters. HOX genes upregulated in PDAC in Yan et al. [14] and Mao et al. [15] studies are indicated in red. **c** HOX genes upregulated in fetal pancreas in red. **d** HOX gene expression signatures in PDAC subtypes. Genes upregulated or downregulated are indicated in red or blue, respectively

Previous studies have shown that expression of developmental regulators is reinitiated in PDAC [10]. RNA sequencing of 16 fetal pancreata (7–14 weeks post conception) was performed to identify novel genes critical for embryonic pancreas development. To identify key genes associated with PDAC, we selected two independent studies [16, 17] that reported gene expression signatures and pathways in this disease. Yan et al. identified 2735 differentially expressed genes (DEGs) in PDAC tissue, with 557 upregulated genes. Similarly, Mao et al. found 2725 DEGs (1554 were upregulated). 332 genes were differentially expressed in both studies (Fig. 1a).

To identify the intersect of genes which were upregulated in both PDAC and fetal tissue, gene expression data from embryonic pancreas was first compared with expression data from adult pancreas (GTEX) to determine genes that were enriched in pancreas anlagen. 327 genes were expressed in fetal pancreas (221 genes with elevated expression of which 69 were exclusively expressed in fetal pancreas).

Next, we assessed if genes enriched in embryonic pancreas and PDAC clustered in specific PDAC subtypes [9] with the majority of the genes belonging to squamous (60) and ADEX (53) subtypes (Fig. 1a, listed in Table S1). Many of these genes have been studied in the context of PDAC biology (Table S1) confirming the validity of our approach. Several DEGs belong to the *HOX* family of transcription factors (Fig. 1b) and have been studied in PDAC [18–20]. However, the functions of *HOXB6* (enriched in progenitor PDAC subtype, Fig. 1b-d) and the closely related [21] *HOXB8* transcription factor (differentially expressed in progenitor and immunogenic PDAC subtype) have not been investigated in PDAC so far.

### *HOXB6* and *HOXB8* are expressed in embryonic mesenchymal and malignant ductal cells

**Fig. 2:**
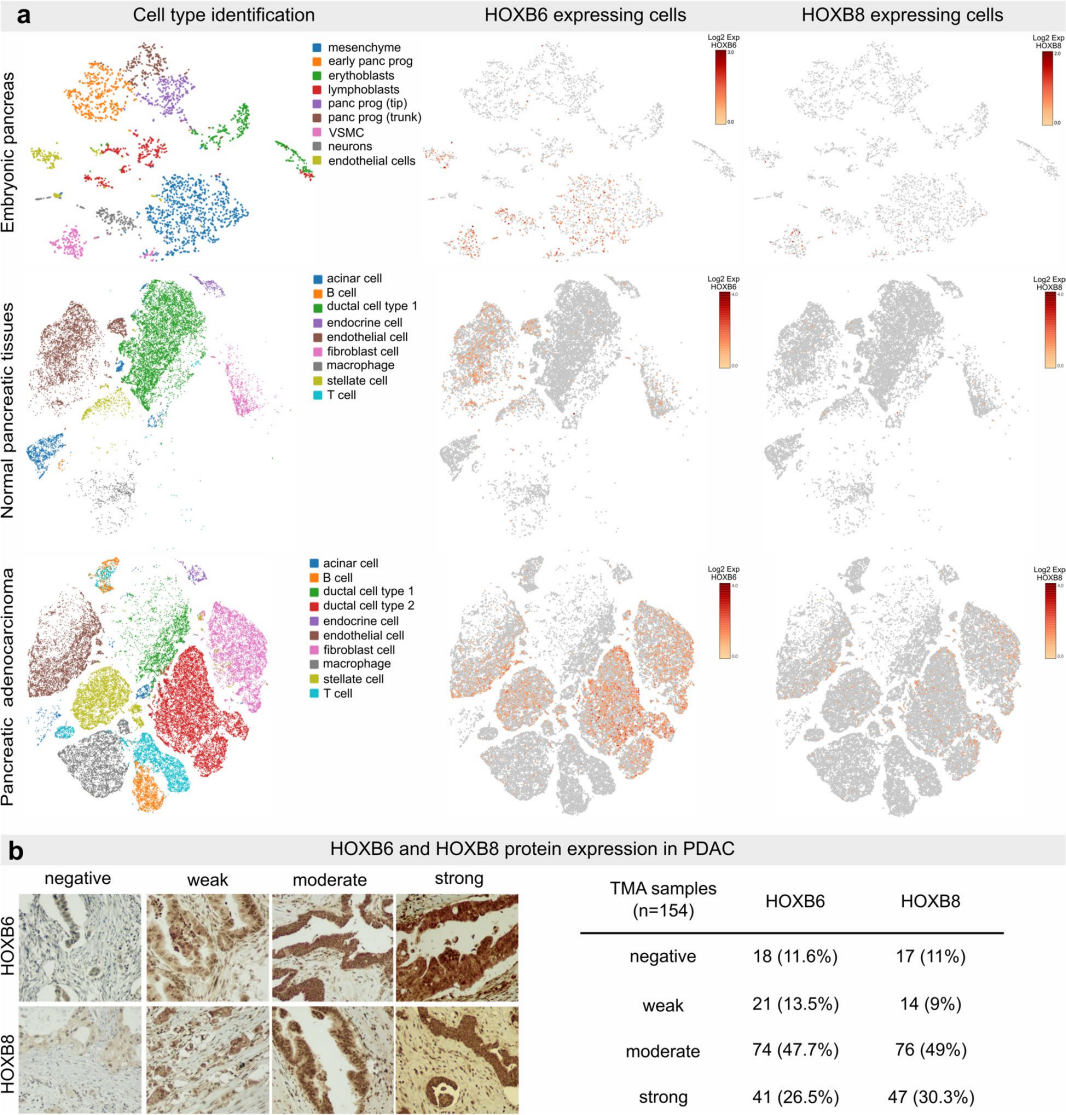
HOXB6 and HOXB8 are expressed in embryonic mesenchyme and malignant ductal cells. **a** t-SNE embedding and HOX gene expression in pancreas from an 8-week PC embryo, in normal adult pancreas and PDAC tissues. Cells with HOXB6 or HOXB8 expression were colored according to their expression levels. **b** Representative images of HOXB6 and HOXB8 immunohistochemical staining and distribution of TMA-samples according to staining intensity (*n* = 154). Original magnification: 200x

Previous studies have detected *Hoxb6* in mouse embryonic mesenchymal cells [22] and adult fibroblasts [23]. HOXB8 expression analysis in the pancreas has not been reported yet. We assessed *HOXB6* and *HOXB8* expression in developing human pancreas and PDAC tissue using scRNAseq [24, 25]. During pancreas development, *HOXB6* was predominantly expressed in mesenchymal cells, whereas *HOXB8* was only present in a few mesenchymal cells and in vascular smooth muscle cells (Fig. 2a). In the adult pancreas, *HOXB6* expression was detected mainly in fibroblasts, endothelial and stellate cell populations (3.8% of all cells), while *HOXB8* expression was limited (0.18% of all cells). In contrast, *HOXB6* transcripts were abundant in PDAC tissues (12% of all PDAC cells). mRNA expression was mainly detected in malignant ductal (23% ductal cell type 2), endothelial (20%) and fibroblast cells (16%). *HOXB8* was expressed in the same cell clusters (2.8%, 2.5% and 3.5% *HOXB8*^+^ cells, respectively, Fig. 2a).

Tissue microarray analysis (TMA) was performed to confirm gene expression and assess if HOXB6 and/or HOXB8 expression were correlated with clinical parameters and patient survival. HOXB6 and HOXB8 protein was detected in most TMA-samples (88.3 and 89% respectively, Fig. 2b). Kaplan–Meier analysis revealed that low HOXB6 expression was associated with poorer disease specific survival compared to moderate expression or high expression (Fig. S1, Table S2). These findings show that HOXB6 and HOXB8 proteins are expressed in the vast majority of all PDAC samples and that variations in protein expression are associated with altered survival rates.

To determine which gene networks were co-expressed with *HOXB6* and/or *HOXB8* in PDAC cells, an unbiased co-expression analysis of single fetal and normal adult cells was performed (Table S3). *HOXB6* and *HOXB8* expression in fetal and PDAC tissue was correlated with genes regulating RNA and protein metabolism, DNA replication, signal transduction, programmed cell death, cell cycle regulation, and stem cell biology (Table S4). These findings suggest that a large subset of *HOXB6* and *HOXB8* expressing cells are pancreatic cancer stem cells and that *HOXB6* and/or *HOXB8* regulate stemness, proliferation, and apoptosis in PDAC cells.

### Reduced *HOXB6* and *HOXB8* expression affects clonogenic capacity, cell proliferation, and viability of PDAC cells

Co-expression analysis showed that *HOXB6* and *HOXB8* expression correlate with genes regulating cell cycle, proliferation, and apoptosis. To determine if these observations were functionally relevant in PDAC cells, we knocked down (KD) HOXB6 and/or HOXB8 expression using siRNAs (i.e., siHOXB6, siHOXB8, and siHOXB6B8) in the human pancreatic cancer cell lines PANC-1 and AsPC1. PANC-1 and AsPC1 cells have been isolated from pancreatic carcinomas of ductal cell origin and highly express HOX genes. siHOXB6, siHOXB8, and siHOXB6B8 KDs were performed with several different siRNAs and confirmed by RT-qPCR and Western blot analyses (Fig.S2).

**Fig. 3:**
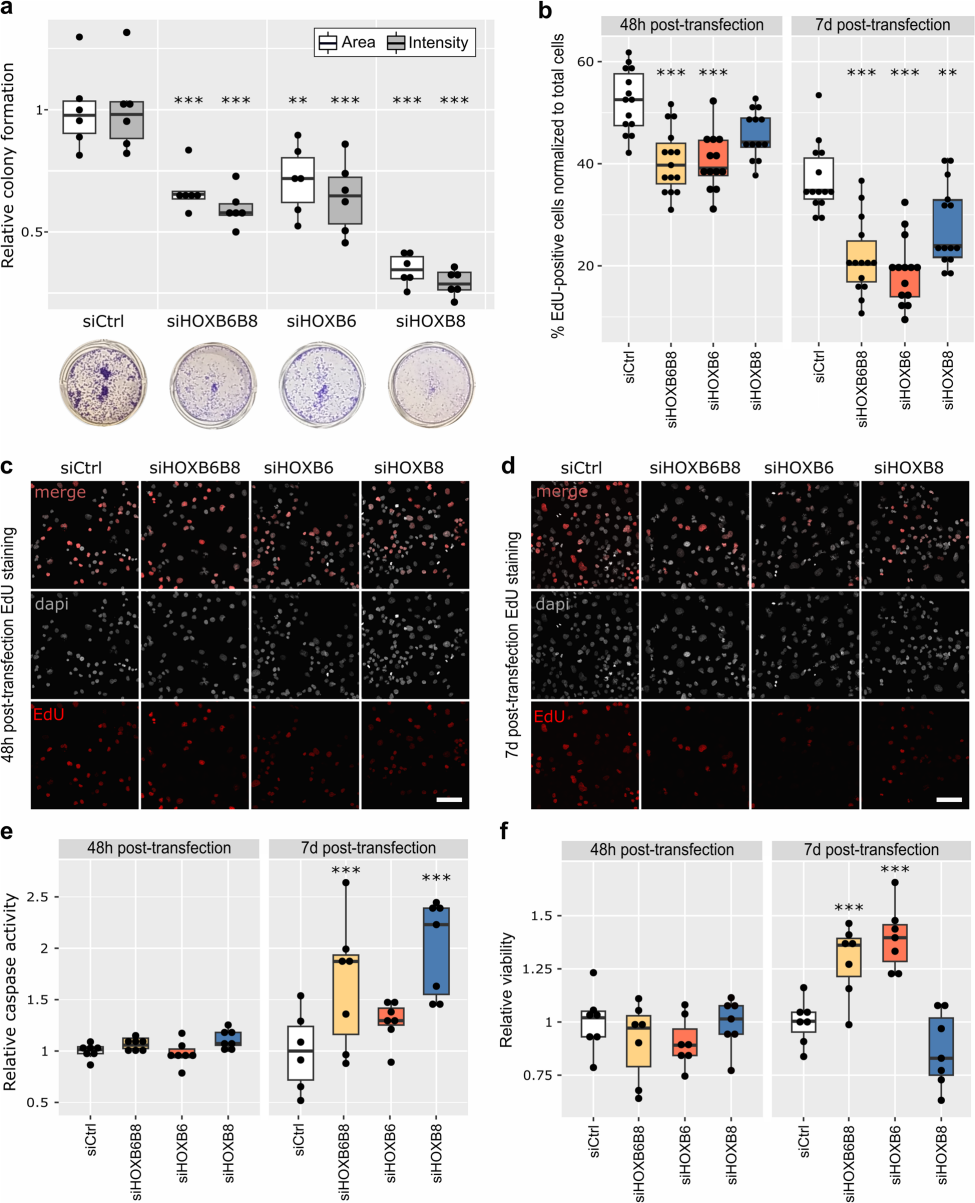
Colony formation, proliferation, cell viability, and apoptotic capacities are impaired in siHOXB6 and siHOXB8 transfected cells. **a** Relative colony formation quantification (*n* = 6). Results are shown as colony area or staining intensity percent 7 days post knock-down compared to negative control (scrambled RNA, siCtrl). **b** Quantification of EdU positive cells (*n* = 14). Results are shown as percent EdU positive cells of total cell number. Dapi and EdU staining 48 h (**c**) or 7 days (**d**) after transfection. Scale bars = 100 µm. Cells were exposed to EdU for 4 h. **e** Relative caspase activity 48 h or 7 days after transfection. Results are shown as caspase-3/7 activity compared to negative control (*n* = 6). **f** Cell viability 48 h and 7 days post transfection. Results are shown as viability/cytotoxicity ratio compared to negative control (*n* = 6). (**a**-**b**, **e**–**f**) Tukey’s post-hoc test significances are indicated by stars compared to control when significant. * *p* < 0.05, ** *p* < 0.01 *** *p* < 0.001

First, we examined if reduced *HOXB6* and *HOXB8* expression affected clonogenic capacity of tumor cells by performing colony formation assays. 7 days post transfection, the area and density of PANC-1 colonies were significantly decreased in all KD conditions compared to the control: in mean, colony formation was reduced by 35% in siHOXB6B8, 30% in siHOXB6, and 65% in siHOXB8 (Fig. 3a).

In order to assess if siHOXB6 and/or siHOXB8 impaired proliferation of tumor cells, EdU incorporation was assessed as a measure of DNA synthesis (S-phase). At 48 h post transfection, the proportion of EdU^+^ cells was significantly decreased in siHOXB6B8 and siHOXB6 (Fig. 3b, c). Cell proliferation was also tested 7 days post transfection to assess long-term effects of siHOXB6 and siHOXB8. EdU^+^ cell number was reduced by 41% in siHOXB6B8, 48% in siHOXB6, and 24% in siHOXB8 cells (Fig. 3b, d).

Colony formation of siHOXB8 cells was significantly more reduced compared to siHOXB6 and siHOXB6B8. These differences in colony formation between KD conditions cannot solely be explained by differences in the proliferation rate. To investigate the different phenotypes observed in siHOXB6 and siHOXB8, apoptosis and cell viability were assessed using ApoTox-Glo triplex assay. At 48 h post transfection, no significant difference in caspase activity and viability rate was observed (Fig. 3e). 7 days post transfection, caspase activity was significantly higher in siHOXB6B8 (+ 65%) and siHOXB8 (+ 100%) cells (Fig. 3e, Fig.S3a). The apoptosis rate was significantly increased in siHOXB8 compared to siHOXB6 cells suggesting that differences in apoptosis rates resulted in decreased colony formation of siHOXB8 cells.

siHOXB6B8 cells had lower proliferation abilities and similar apoptosis rate, but more colonies than siHOXB8 cells. To assess if these changes were associated with differences in cell viability, Triplex assays were performed 7 days post transfection. siHOXB6 (+ 39%) and siHOXB6B8 (+ 28%) cells showed significantly increased cell viability whereas cell viability wasn´t affected in siHOXB8 (Fig. 3f, Fig.S3b). These findings show that cell viability is specifically affected by *HOXB6* function while *HOXB8* had a larger effect on apoptosis.

### Reduced *HOXB6* and *HOXB8* expression enhances senescence and gemcitabine sensitivity

**Fig. 4:**
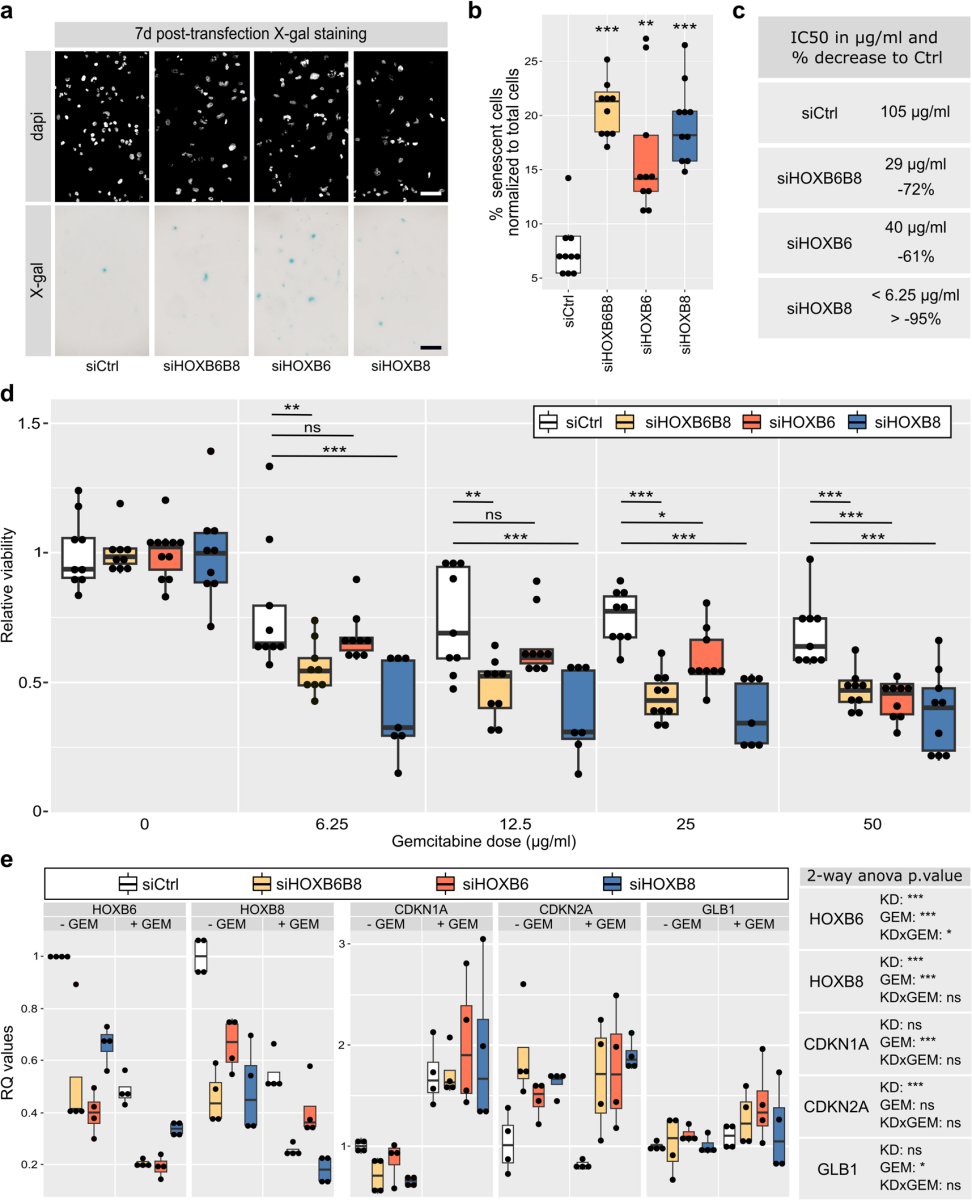
HOXB6 and HOXB8 regulate cell senescence and gemcitabine sensitivity. **a** Senescence-associated galactosidase activity for siHOXB6, siHOXB8, and siHOXB6B8 is shown in blue. Scale bar = 100 µm. **b** Quantification of senescent cells for all conditions as percent per total cell number (*n* = 10). **c** Quantification of the concentrations of gemcitabine required to reduce cell number by 50% (IC_50_) from the (**d**) Relative viability of 7-days transfected cells treated with varying concentrations of gemcitabine (*n* = 9)*.* MTT assay quantifying the relative cell viability after 6 h-treatment with varying concentration of gemcitabine (µg/ml) to the dose 0 for each transfected cell condition. **e** Quantification of HOXB6, HOXB8, and ageing marker expression of 7-days transfected cells with or without 50 µg/ml of gemcitabine treatment (*n* = 4). 2-way ANOVA p.value results are indicated. KD: knock-down variable, GEM: gemcitabine treatment variable, KDxGEM: interaction effect between the knock-down and the gemcitabine treatment variables. Tukey’s post-hoc test significances are indicated by stars compared to the control when significant. ns *p* > 0.05, * *p* < 0.05, ** *p* < 0.01 *** *p* < 0.001

Decreased proliferation rates and colony formation, but increased cell viability of siHOXB6 and siHOXB6B8 PDAC cancer cells may result from altered cellular senescence which is characterized by stable viability with resistance to apoptosis [26]. siHOXB6 and siHOXB8 cells were assessed for galactosidase activity as a measure of cellular senescence. At 7 days post transfection, siHOXB6 (+ 107%), siHOXB8 (+ 137%) and siHOXB6B8 (+ 159%) cells displayed significantly higher galactosidase activity than control cells (Fig. 4a, b). This indicates that HOX KD cells become senescent more readily.

Senescence of cancer cells is associated with treatment resistance [27, 28]. To determine if the increased cellular senescence of KD cells was linked to pancreatic cancer treatment resistance, we assessed sensitivity to gemcitabine, the primary pancreatic cancer treatment. In a dose–response experiment with Gemcitabine, siHOXB6 and/or siHOXB8 cells were more sensitive to treatment (Fig. 4d). The concentrations of gemcitabine required to reduce cell number by 50% (IC_50_) were significantly lower for KD cells (Fig. 4c) suggesting that *HOXB6* and *HOXB8* function in PDAC cells contributes to gemcitabine resistance.

Gene expression analysis showed that gemcitabine treatment reduced *HOXB6* and *HOXB8* mRNA levels significantly (Fig. 4e), and expression was even further reduced in gemcitabine-treated KD cells. siHOXB6 and siHOXB8 cells had increased cyclin-dependent inhibitor *CDKN2A* expression, which is essential for growth arrest in senescent cells. Enhanced *CDKN2A* expression was not induced by gemcitabine treatment alone, suggesting that HOXB6 and HOXB8 regulate *CDKN2A* (and cellular senescence) independently of gemcitabine’s actions. Interestingly, *CDKN1A* expression was only increased in gemcitabine-treated cells (Fig. 4e) indicating that this part of senescence signaling (in contrast to *CDKN2A*) is independent of *HOXB* gene expression.

These findings show that *HOXB6* and *HOXB8* regulate senescence escape and gemcitabine resistance in PDAC.

### *HOX* genes regulate cell cycle, proliferation and senescence pathways

*HOXB6* and *HOXB8* transcription factors have multiple roles in gene regulation [29]. To assess how HOXB6 and/or HOXB8 transcriptional activity affects gene expression in pancreatic cancer cells, RNA sequencing of siHOXB6 and/or siHOXB8 PANC-1 cells was performed. Analysis of siHOXB6, siHOXB8, and siHOXB6B8 samples indicated altered expression for 5611, 5905, and 7384 genes, respectively (Table S5). 2553 common target genes were dysregulated in all conditions.

**Fig. 5:**
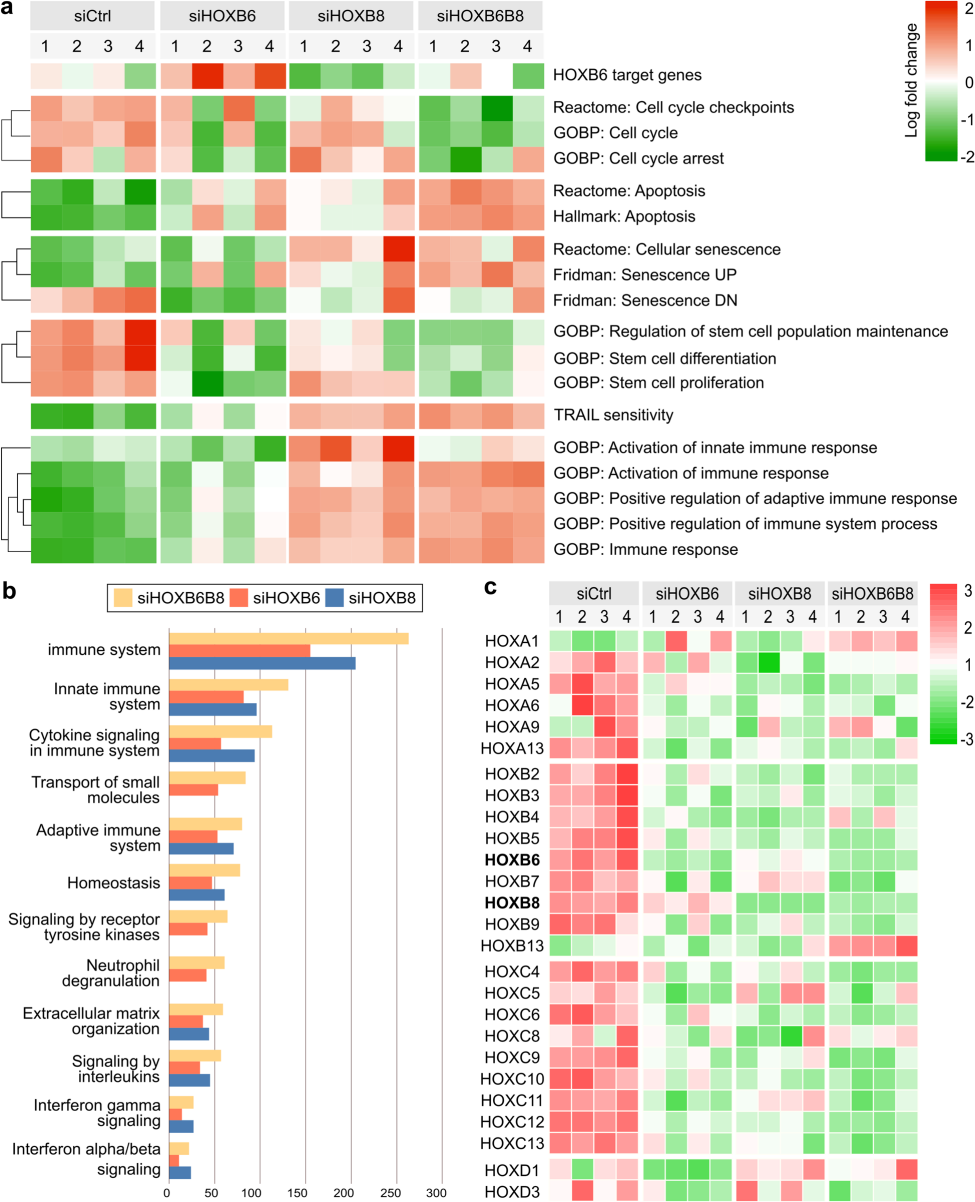
Transcription profiles and pathway analyses siHOXB6, siHOXB8, and siHOXB6B8 in PDAC. **a** Gene set enrichment analyses (*n* = 4). **b** Number of genes identified in Reactome enrichment pathways up-regulated in siHOXB6, siHOXB8, and siHOXB6B8. **c** Significantly altered HOX gene expression in siHOXB6, siHOXB8, and siHOXB6B8

To identify novel cellular processes and functions associated with *HOXB6* or *HOXB8* expression, DEGs were analyzed using MSigDB [30, 31], Gene set enrichment analyses (GSEA), Reactome (reactome.org [32]), and STRING database [33]. GSEA analyses showed that HOXB6 and/or HOXB8 regulated cell cycle, apoptosis, cellular senescence, and stem cell differentiation pathways (Fig. 5a). Stem cell marker gene expression was reduced in all experimental conditions (Fig.S3c) which was also observed in AspC1 cells (Fig.S3d), confirming that HOXB6 and HOXB8 regulate these processes in PDAC cells.

RNA-seq analysis of siHOXB6 and siHOXB8 showed that HOXB6 and HOXB8 mainly affected the same cellular processes/signaling pathways (Fig. 5b). However, for several gene sets, the effect of either *HOXB6* or *HOXB8* KD was more pronounced and double KD samples had strongest changes in gene expression (Fig. 5a). For example, expression of genes associated with immune response was more increased in siHOXB8 and siHOXB6B8, while stem cell and cell cycle gene sets were more down regulated in siHOXB6 and siHOXB6B8. Interestingly, HOXB6 and HOXB8 regulate expression of multiple *HOX* genes (Fig. 5c). STRING functional enrichment analyses using Reactome pathways (Fig. 5b) confirmed GSEA observations and showed an over-representation of genes regulating the immune system in KD cells, suggesting that *HOX* genes promote tumor cell survival through promoting immune evasiveness.

**Fig. 6:**
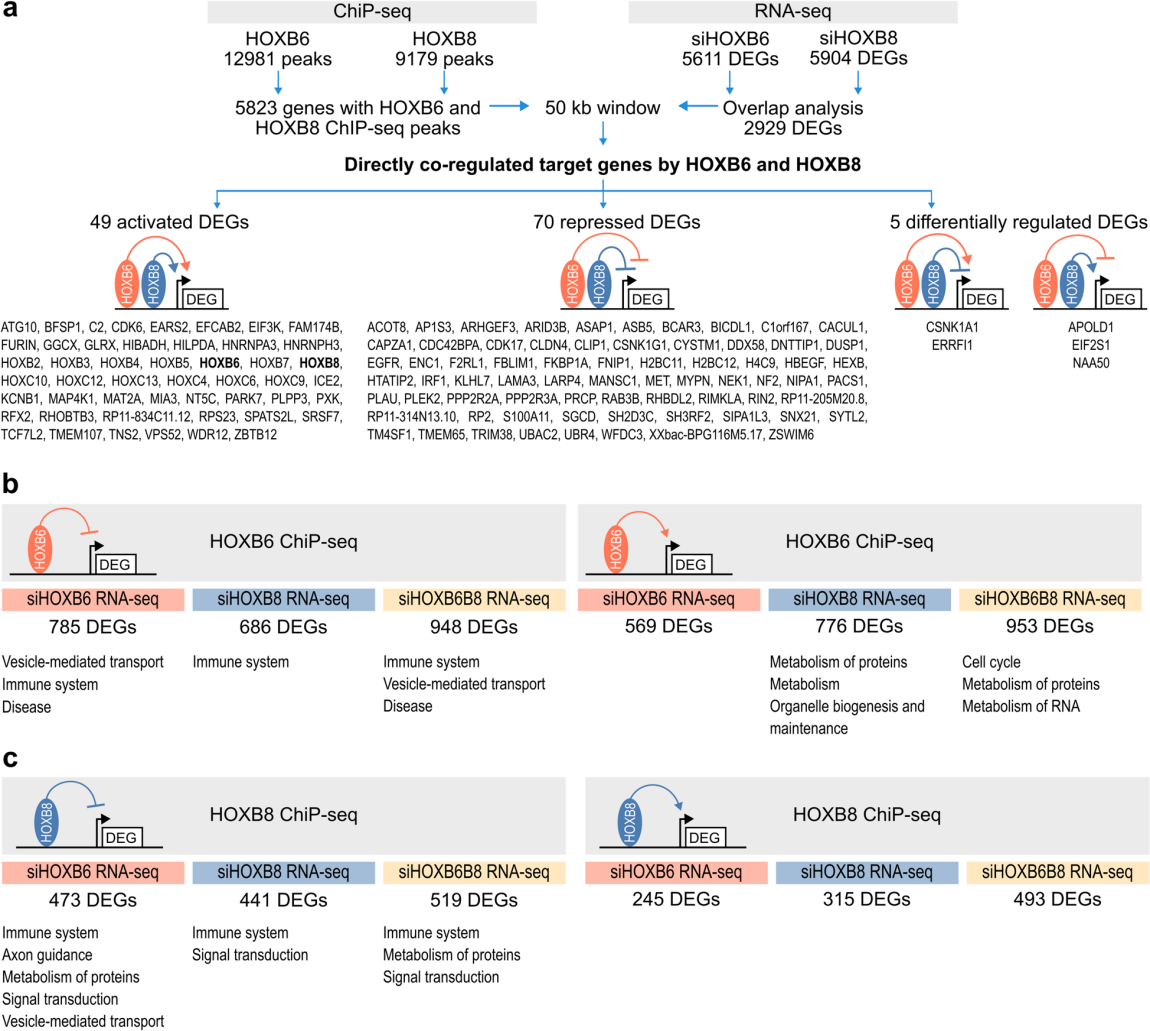
Integrated chromatin occupancy and gene expression analysis of HOXB6 and HOXB8 in PANC-1 cells. **a** Strategy for integrating HOXB6 and HOXB8 ChIP-seq and RNA-seq data to identify common direct target genes and pathways. **b** HOXB6 target genes differentially regulated in siHOXB6, siHOXB8, and siHOXB6B8 RNAseq. **c** HOXB8 target genes differentially regulated in siHOXB6, siHOXB8, and siHOXB6B8 RNAseq. For each ChIP-seq and RNA-seq dataset interaction, the list of the Reactome pathways of the identified DEGs is shown

To identify which genes and pathways were directly regulated by HOXB6 and/or HOXB8, the differentially expressed gene lists were compared with HOXB6 and HOXB8 chromatin immunoprecipitation data (ChIP-seq) in PANC-1 [34] and colorectal cells [35]. In this analysis, more than 5000 genes had binding sites for both HOXB6 and HOXB8 and 2929 genes were differentially regulated in siHOXB6 and siHOXB8 (Fig. 6a). 49 genes were directly bound and activated by HOXB6 and HOXB8 including *HOXB6* and *HOXB8* among other *HOX* genes (Fig. 6a). 77 genes were bound by both HOXB6 and HOXB8 and expression was repressed, while only 5 genes were differentially regulated by HOXB6 and HOXB8.

Most of the HOXB6 and HOXB8 target genes had increased expression in KD cells, suggesting that HOXB6 and HOXB8 act predominantly as repressors in PANC-1 cells (immune system target genes) (Fig. 6b, c). In addition, HOXB6 bound to and activated expression of cell cycle genes (Fig. 6b). As HOXB6 promoted transcription of HOXB8, there was a considerable portion of DEGs in HOXB6 KD cells that had binding sites for HOXB8 (Fig. 6c) and were also differentially regulated in double KD cells. Similarly, *HOXB6* expression was activated by HOXB8 and HOXB6 target genes were differentially expressed in HOXB8 KD cells (Fig. 6b) further illustrating that HOXB6 and HOXB8 regulate the expression of one another and act on the same gene sets in PDAC cells.

### HOXB6 and HOXB8 modulate immune responses

siHOXB6 and siHOXB8 RNAseq analysis suggested that *HOX* genes regulate the transcription of genes associated with cellular immune response and possibly tumor immune evasiveness. To assess if HOXB6 and HOXB8 affect tumor cell-immune interactions in vivo the correlation between *HOX* gene expression and estimated immune cell infiltration was assessed using TIMER algorithms (http://timer.cistrome.org/ [36–38]).

**Fig. 7:**
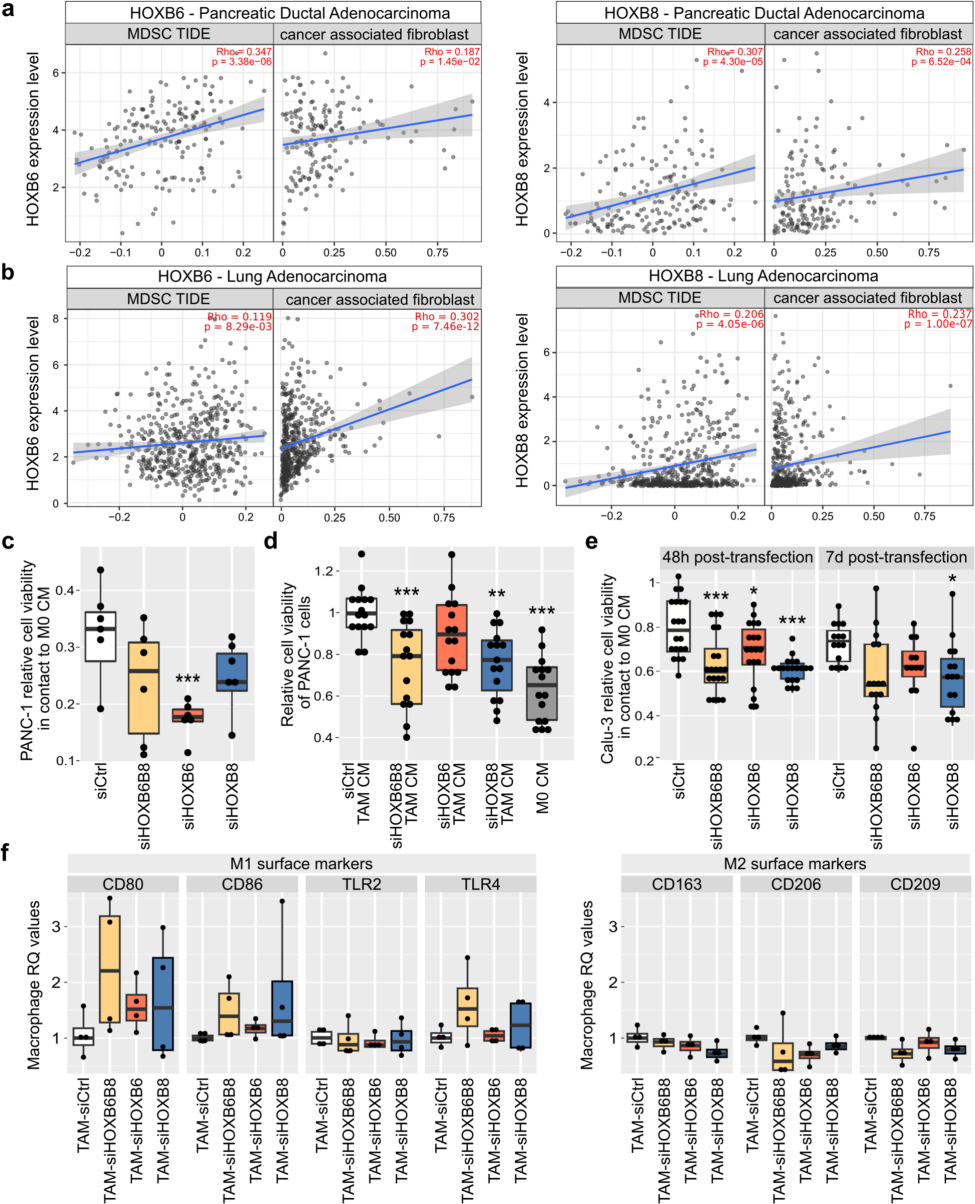
HOXB6 and HOXB8 modulate immune-cancer cell interactions. Correlation of HOXB6 and HOXB8 expression (log2 TPM) with the infiltration level of myeloid-derived suppressor cells (MDSC) and cancer associated fibroblasts estimated by TIMER [36–38] in PDAC (**a**) and LUAD (**b**) (*n* = 179). Relative viability of PANC-1 cells (**c**, **d**) or Calu-3 cells (**e**) treated with macrophage conditioned medium (CM). MTT assays quantifying the relative cell viability of PANC-1 transfected cells (**c**, 7 days post transfection, *n* = 6) or Calu-3 transfected cells (**e**, 48 h (*n* = 20) or 7 days post transfection (*n* = 15)) after treatment with M0 CM compared to associated untreated transfected cells and (**d**) of PANC-1 cells exposed to conditioned media from siHOX-specific TAMs compared to PANC-1 cells exposed to siCtrl TAM CM (*n* = 15). **f** Quantification of the expression level of M1 and M2 surface markers from siHOX-specific TAMs (*n* = 4). Tukey’s post-hoc test significances are indicated by stars compared to the control when significant. * *p* < 0.05 ** *p* < 0.01 *** *p* < 0.001

The TIMER immune association module showed that *HOXB6* and *HOXB8* expression was positively correlated with the presence of immunosuppressive cell infiltrates such as myeloid-derived suppressor cells (MDSC) and tumor-associated fibroblasts (Fig. 7a, *n* = 179) in PDAC samples, further supporting an association between *HOX* gene expression and immune response in PDAC.

To assess if *HOXB6* and/or *HOXB8* expression influenced immune cell-pancreatic tumor interactions, co-culture experiments of siHOXB6 and siHOXB8 PANC-1 cells with differentiating THP-1 monocytes were performed. THP-1 monocytes were stimulated with PMA to induce differentiation to M0 macrophages and conditioned medium (CM) was collected. The cellular response of siHOXB6 and siHOXB8 PANC-1 cells to M0 CM (see Fig.S4 for an overview of the experimental design) was assessed. As expected, exposure to M0 CM decreased cell number in siCtrl cells, HOX KD cell numbers decreased even further with siHOXB6 cells being the most sensitive (-45% compared to siCtrl + M0) to anti-tumorigenic proprieties of M0 macrophages (Fig. 7c).

Tumor environment promotes the polarization of M0 macrophages into tumor-associated macrophages (TAMs) which can produce pro-inflammatory and anti-tumor signals (M1 macrophages) or anti-inflammatory and pro-oncogenic signals (M2 macrophages) and promote tumor cell survival [39, 40]. Polarized macrophages can switch between phenotypes depending on the tumor microenvironment [41]. To test if *HOXB6* and/or *HOXB8* contribute to the polarization of M0 macrophages, M0 macrophages were co-cultured with control and HOX KD cells (Fig.S4b), CM of TAMs was collected, PANC-1 cells treated with TAM-CM, and cell viability assessed. Control CM promoted pro-tumoral TAM polarization and subsequently PANC-1 cell viability (Fig. 7d). In contrast, CM from siHOXB8 and siHOXB6B8-TAMs significantly decrease cell viability of PANC-1 cells (-24 and -26%, respectively) (Fig. 7d). qPCR analysis from TAM RNA was performed determine if macrophage polarization was affected by co-culture with HOX KD cells (Fig. 7f). Co-culture with KD PANC-1 cells increased expression of M1 marker genes, while M2 marker gene expression was slightly decreased showing that M0 macrophages co-cultured with HOX KD cells maintained their capability to promote tumor cell death by adapting a more M1 macrophage-like phenotype.

To determine if *HOXB6* and/or *HOXB8* regulate the immune response specifically in PDAC, we performed a TIMER association analysis of *HOXB6* and *HOXB8* expression with all TCGA cancer samples currently included in this analysis module. From the 40 cancer types studied, *HOXB6* (Fig.S5) and *HOXB8* (Fig.S6) expression correlated with cancer-associated fibroblast and myeloid-derived suppressor cell infiltration in 7 different types of cancer. From this list, only the lung carcinoma (LUAD) had a similar correlation pattern between *HOXB8* expression and monocyte and macrophage infiltration (Fig. 7b, S6). Therefore, we assessed if *HOX* genes controlled macrophage-cancer cell interactions in Calu-3 cells, a LUAD cell line.

The sensitivity of siHOXB6 and/or siHOXB8 Calu-3 cells to exposure with M0 CM (Fig.S4C) was determined. As observed for PANC-1, exposure to M0 CM decreased cell number and HOX KDs cells were more sensitive to the anti-tumorigenic proprieties of M0 macrophages (-20%, -15%, and -21% for 7-day siHOXB6B8, siHOXB6, and siHOXB8, respectively (Fig. 7e)). However, high variations were observed between replicates due to variable KD efficiencies 7 days post transfection (Fig.S7). KD efficiency was more consistent 48 h post transfection (Fig.S7) and cell viability of HOX KD Calu3 cells was reduced upon M0 CM exposure (-21%, -14%, and -24% for 48 h siHOXB6B8, siHOXB6, and siHOXB8, respectively (Fig. 7e)). We also assessed if Calu-3 cells contributed to the differentiation of M0 macrophages to TAM. Our results showed that M0 macrophages were losing their anti-tumorigenic proprieties upon incubation with Calu-3 cells and that the collected medium did not affect Calu-3 viability (Fig.S7), suggesting that Calu-3 cells were not responding to Calu-3 TAM CM.

Our data show that *HOX* genes promote PDAC cell-induced differentiation of TAM, as well as PDAC and LUAD cell resistance to M0 macrophages suggesting that elevated *HOXB6* and *HOXB8* expression levels contribute to an immune microenvironment that promotes enhanced tumor cell survival in different cancer types at least in vitro.

## Discussion

*Hox* genes are well characterized transcription factors that establish the anterior–posterior axis in vertebrates and are critical for the morphogenesis of multiple organs [42]. Gene duplication has resulted in the presence of several different *HOX* clusters (A to D) with paralog groups expressed in overlapping domains having similar DNA binding abilities. In addition to their roles in development, aberrant expression has also been associated with tumor formation and *HOX* genes regulate tumor cell proliferation, growth, migration, and apoptosis [15]. In these instances both loss and gain of *HOX* gene expression may promote tumorigenesis and the same *HOX* gene can act as tumor suppressor or proto-oncogene depending on the cellular context [43]. In PDAC, aberrant expression of several HOX transcription factors has been described and functional studies have shown that *HOXA10* [20], *HOXB7* [19], and *HOXC11* [44] promote pancreatic cancer development while *HOXA1* [45], *HOXB1* [46], and *HOXB3* [18] act as tumor-suppressors. However, the roles and interaction of *HOXB6* and *HOXB8* in PDAC have not been studied extensively. *HOXB8* has been described as a target of the microRNA miR-2682-5p in a signaling cascade involving LINC01006-miR-2682-5p-HOXB8 that promotes pancreatic cancer proliferation and metastasis [47], while *HOXB6* expression has been reported previously in mouse pancreatic fibroblasts which were not associated with the tumor stroma [23]. Our expression analysis showed that, although abundant expression was observed in embryonic mesenchymal cells, *HOXB6* was predominantly expressed in malignant ductal cells in PDAC while *HOXB8* expression was limited to few cells during pancreas development and expression was virtually absent in adult pancreas but initiated in PDAC ductal cells suggesting that upregulation of these genes contribute to tumor formation.

Gene expression analysis of *HOXB6* and *HOXB8* KD cells suggested that HOXB6 and HOXB8 promote PDAC tumorigenesis, but despite of having high molecular similarities the cellular mechanisms involved differ: HOXB6 more strongly promotes expression of genes important for stem cell differentiation whereas HOXB8 and HOXB6 repress expression of genes critical for immune-cancer cell interactions. Interestingly, HOXB6 and HOXB8 bind to and activate expression of one another and other *HOX* genes expressed in pancreatic cancer cells suggesting that a HOX transcriptional network is active in PDAC (Fig. 5) similarly to what has been described in other solid tumors (reviewed in [48, 49]).

Single cell RNAseq analysis showed that *HOXB6* expression is present in malignant ductal cells, a cell population that also includes PDAC stem cells. A previous transcriptional characterization of PDAC stem cells showed that these cells have abundant *HOXB6* expression suggesting that HOXB6 function promotes tumor stem cells [50]. Most importantly, we show that *HOXB6* and *HOXB8* KD impaired tumor cell proliferation, differentiation, and maintenance further supporting that HOXB6 and HOXB8 are important regulators of pancreatic cancer stem cells. *HOXB6* correlated with *BMI1* and *SOX9* expression levels and previous studies have shown that SOX9 enhances tumor progression through *BMI1* binding and p21 down-regulation which induces cell proliferation and evasion of apoptosis and senescence [51]. These findings are similar to our observation that *BMI1* expression is down-regulated in siHOXB6 and siHOXB6B8 cells which correlates with the induction of senescence, repression of tumor cell proliferation, and up-regulation of the tumor suppressor *CDKN2A*. Moreover, *HOXB6* and *HOXB8* expression is decreased in gemcitabine-treated PDAC cells and expression levels affected PDAC cell sensitivity to gemcitabine treatment further supporting that *HOX* genes act as key regulators to prevent tumor senescence and treatment efficacy in PDAC.

In addition, siHOXB8 cells had enhanced expression of death receptor genes which control sensitivity of tumor cells to TRAIL, a cytokine produced in peripheral tissue, to promote tumor cell death [52]. Specifically, increased expression of the trail receptor genes *TNFRSF10B* and *TNFRSF10A* was observed in siHOXB6 and siHOXB8 cells. Previous studies have shown that low levels of TRAIL receptors contribute to worse prognosis, while the presence of cytoplasmic TRAIL-R1 is a positive prognostic marker for PDAC patients [53, 54] and a combined treatment of gemcitabine with gene-modified mesenchymal stem cells expressing TRAIL shows promising results in treating PDAC [55]. Our findings suggest that HOXB6 and HOXB8 modulate treatment resistance in PDAC by regulating cell senescence, gemcitabine response, and TRAIL expression.

A categorization of genes highly expressed in PDAC and embryonic pancreas, showed that a large portion of genes (including *HOXB6*) were associated with the pancreatic progenitor PDAC subtype, while *HOXB8* expression was associated with pancreatic progenitor and immunogenic subtypes. The existence of an immunogenic PDAC has been discussed, and it has been suggested that the immune cell expression signature in this subtype is due to contamination by infiltrating B and T cells, while a gene expression pattern similar to pancreatic progenitor-like cells is also present [56]. We observed impaired cell proliferation and colony formation in siHOXB6 PANC-1 cells which is in line with elevated HOXB6 expression in the pancreatic progenitor PDAC subtype. siHOXB8 PANC-1 cells exhibited impaired cell proliferation, but also suppressed induction of tumor-associated macrophages illustrating that HOXB8 contributes to both the pancreatic progenitor and immunogenic features observed in the immunogenic PDAC subtype.

Medium conditioned by naïve M0 macrophages affected cell viability of siHOXB6 PANC-1 and siHOXB6/8 Calu-3 lung cells and co-culture experiments of siHOXB8 cells with differentiating macrophages showed that reduced *HOXB8* expression directed tumor-associated macrophages towards a M1 polarization state. This illustrates that HOXB8 has a critical role in tumor-immune cell interaction by modulating macrophage differentiation towards a more tumor-supportive cell state. Correlation analysis between *HOX* gene expression and immune cell infiltration in human PDAC and LUAD tissues show that these mechanisms are also present in vivo in at least two different adenocarcinoma types that had relatively high *HOXB8* expression and co-culture experiments performed in LUAD and PANC-1 showed that the regulation of immune-cancer cell interaction of *HOX* genes is conserved. Moreover, a recent publication showed that *HOX* genes control similar processes in endometrial cancer with a *HOX* expression score being proposed to predict among other parameters the efficacy of immunotherapy [57]. This suggests that *HOXB6* and *HOXB8* expression (in at least pancreatic and lung adenocarcinoma) enhance immunosuppressive effects of cancer cells by promoting immune evasiveness of macrophages and thereby contributing to an immunosuppressive microenvironment affecting macrophage differentiation.

In this study, we have focused on analyzing HOXB6 and HOXB8’s effects on pancreatic (and lung) adenocarcinoma in vitro, we also used publically available data from a larger collection of tumor samples to verify gene expression, immune cell infiltration, and correlate to patient outcomes. Cell culture experiments have several advantages but also limitations. For example, tumor cell behavior (proliferation, apoptosis, senescence) in response to reduced *HOXB6* and *HOXB8* expression could be observed and co-culture experiments with M0 macrophages allowed us to study if *HOXB6* and *HOXB8* are important for immune-cancer cell interactions. However, to establish a critical role for *HOXB6* and *HOXB8* in pancreatic cancer biology, animal models and primary human PDAC cell culture will be needed to verify our findings in an in vivo setting. These experiments will also be critical in investigating how HOXB6 and HOXB8 influence gemcitabine treatment efficiency.

In conclusion, our findings indicate that *HOX* genes play an important role in regulating cell proliferation, cell death, immune response, and treatment resistance in pancreatic cancer cells. These results are consistent with the pathways correlating with *HOXB6* and *HOXB8* in PDAC tissues and the immune cell infiltrates observed in PDAC and LUAD. Our data suggest that *HOXB6* and *HOXB8* regulate numerous oncogenic pathways to promote adenocarcinoma tumorigenesis.

## Materials and methods

### Gene expression profiling in human fetal tissues and comparison with adult pancreas

Embryonic pancreata were obtained from terminated fetuses (7–14 gestational weeks, *n* = 16). Informed consent was obtained from the participating women.

RNA was extracted for RNAseq, libraries generated, and comparison of fetal-specific gene expression with adult pancreas expression was performed. For comparison with adult tissues, data from GTEX was used. Raw data (bam files) were downloaded from the GTEX database for comparison with fetal datasets (accessed first in April 2019, updated in August 2022). To ensure consistency in data processing, the RNAseq expression quantification pipeline from GTEX V8 (https://github.com/broadinstitute/gtex-pipeline/) was used. Paired-end 101 bp-long reads were aligned to the Reference Human Genome Build 38 using STAR v2.5.3a, to the reference genome annotation Gencode v26. After alignment and post-processing, expression quantification was performed following the pipeline using RSEM and RNASeqQC, resulting in count matrix normalized for library sizes. Normalization of library sizes was performed in edgeR by dividing counts by the library counts sum and multiplying results by a million to obtain counts per million (CPM) values.

Comparison with expression in adult pancreas: edge-R was used to perform differential expression analysis with age and sex as covariates for the genes of interest. Batch correction was performed using COMBAT.

Single-cell RNAseq of embryonic pancreas was performed and analyzed as previously described [25, 58].

### Gene expression studies in PDAC samples

Sequencing data from PDAC (*n* = 24) and control samples (*n* = 11) from Peng et al. [24] were used (accession number: CRA001160) and processed as closely as possible to the parameters described previously [24]. Ten cell types were identified and categorized among the PDAC samples separately according to the criteria used by Peng et al. [24]. These cells were re-clustered in Loupe browser with default settings to get a tSNE plot, which resulted in a total of 57383 re-clustered cells (PDAC = 41862 and control = 15,521). *HOX* expression was then filtered for, and plots were generated as previously described [58].

### Cells

PANC-1 [59] and THP-1 [60] cell lines were obtained from the European Collection of Authenticated Cell Cultures (Sigma-Aldrich, MO, USA, 87092,802 and 88081201). Calu-3 and AsPC1 were obtained from Darcy Wagner and Roland Andersson (both Lund University), respectively. PANC-1 cells were maintained in DMEM high glucose medium (Sigma-Aldrich, D6429), THP-1 and AsPC1 cells in RPMI-1640 medium (Gibco, 11875–093), Calu-3 cells in DMEM low glucose medium (Gibco, 31885023). Media were supplemented with 10% FBS and 1% penicillin–streptomycin (Gibco, 15140122, PANC1, Calu-3) or 1% penicillin–streptomycin-neomycin (Gibco, 15640055, THP-1, AsPC1). Cells were incubated at 37 °C in a humidified incubator with 5% CO_2_ and frequently tested for mycoplasma contamination using MycoAlert™ detection kit (Lonza, LT07-118).

PANC-1, AsPC1, and Calu-3 cells were transfected with Silencer™ Select negative control siRNA (Invitrogen, USA, 4390843), Silencer™ Select HOXB6 siRNA (Invitrogen, s6805 (siHOXB6) and s6804 (siHOXB6’)) and/or Silencer™ Select HOXB8 siRNA (Invitrogen, s6810 (siHOXB8) and s223867 (siHOXB8’) at 10 nM (PANC-1, Calu-3) or 30 nM (AsPC1) with RNAiMax Lipofectamine (Invitrogen, 13778150) according to the manufacturer’s guidelines for 48 h or 7 days. Transfection efficiency was validated using Silencer™ Cy™3-labeled GAPDH siRNA (Invitrogen, AM4649).

### RNA-seq of PANC-1 cells

Seven days after transfection, RNA was extracted, RNA libraries constructed, and RNA sequencing performed on a NovaSeq 6000 System (Illumina, USA). Data quality assessment was done using FastQC tool and the quality control analysis was performed using the Cutadapt tool (version 4.4). STAR (Spliced Transcripts Alignment to a Reference) tool was used to align reads with human reference genome (hg38). Count data of expressed genes were generated using hg38 annotation gtf file and then subjected to differential gene expression analysis using R package DESeq2. The filtering criteria used for obtaining a list of statistically significant differentially expressed genes was *p*-value < 0.05 and Log2FC > 1 or < -1 (for upregulated or downregulated genes, respectively).

### ChiP-seq analyses

Published HOXB6 [35] and HOXB8 [34] ChIP-seq dataset were compared to PANC-1 RNA-seq results to identify potential direct HOXB6 and/or HOXB8 target genes. STRING functional enrichment analyses (string-db.org [33]) of the identified DEGs were performed to identify significantly enriched Reactome pathways.

### TIMER

Correlations between immune infiltration level and *HOXB6* or *HOXB8* expression in cancer types from the cancer genome atlas program (TCGA) were obtained from TIMER 2.0 web interface using immune association module [36–38] (http://timer.cistrome.org/, accessed first in August 2023, updated in August 2024).

### Study population and tumor tissue microarray analyses

The cohort consisting of 154 patients with pancreatic adenocarcinoma was described previously [58]. Immunohistochemistry of paraffin sections was performed using HOXB6 (219,499, Abcam, England, 1:200) or HOXB8 (bs6339R, Thermo Fischer, USA, 1:200) antibodies. Immunoreactivity in tumor cells was interpreted independently by two researchers (T.K. and J.H), while the researchers were blinded to clinical outcomes.

Negative staining was scored as 0, weakly positive as 1, moderately positive as 2, and strongly positive as 3 separately for tumor cells for both HOXB6 and HOXB8 and overall disease-specific survival (DSS) analyses were performed as described previously [58].

### qPCR

Total RNA was extracted using RNeasy Qiagen kit and cDNA was generated. qPCR assays were performed using StepOnePlus Real-Time PCR System. Relative gene abundance was calculated using the ΔΔCt method and expressed as FC to control. Primers are listed in Table S6. TBP and ribosomal gene S18 were used as housekeeping genes for AsPC1, PANC1, and Calu3, RNA analyses. RPL37A and GAPDH were used as housekeeping genes for tumor-associated macrophage analyses.

### Western blot

Transfected PANC-1 cells were lysed using RIPA buffer with protease and phosphatase inhibitors. Whole-cell extracts (10 μg) were separated in Tris–glycine sodium dodecyl sulphate (SDS) gels and transferred onto polyvinylidenedifluoride (PVDF) membranes. HOXB6 (1:1000, Santa Cruz biotechnology, sc- 166,950) and HOXB8 (1:400, antibodies.com, A43247) primary antibodies were used. Band density for target proteins was normalized against total protein load and analyzed using Image Lab software.

### Colony assay

Colony formation assay was performed as previously described [58] and analyzed by ColonyArea plugin [61] with ImageJ software. Colony formation was quantified by determining the percentage of area covered by cell colonies and the cell density according to the intensity of the staining.

### Proliferation

Proliferation was quantified using Click-iT™ Plus EdU Cell Proliferation Kit for Imaging (Invitrogen, C10640). Cells were prepared as described previously [58] and EdU labelling was done according to manufacturer´s instructions (Invitrogen, C10640).

### Triplex assay

PANC-1 and AsPC1 cells were transfected with siRNAs in 96-well plate and incubated for 48 h or 7 days. ApoTox-Glo triplex assay kit (Promega, WI, USA, G6320) was used according to manufacturer's instructions.

### Senescence

Senescence-associated β-galactosidase staining was done as previously published [62] 7 days post transfection in 8-well chamber slides. Cells were fixed and incubated with freshly prepared SA β-gal stain containing X-gal (Roche, 11680293001) for 16 h at 37 °C in an incubator without CO_2_. Then cells were washed and stained with DAPI. Images were acquired using a slide-scanner microscope (Olympus VS120) and quantification was done using the Cell Counter Plugin in ImageJ to manually tag and count stained cells for each color channel. Blue-colored cells were counted as senescent cells and the ratio to the total cell number determined.

### Gemcitabine

PANC-1 cells were transfected with siRNAs in 96 well plates at a density of 1 × 10^3^ cells/well (siCtrl), 1.5 × 10^3^ cells/well (siHOXB6 and siHOXB6B8) or 3 × 10^3^ cells/well (siHOXB8) to obtain the same cell density 7 days post transfection. 7 days after transfection, cells were exposed to increasing concentration of gemcitabine (Sigma-Aldrich, G6423) for 6 h. Then, cells were washed and maintained in complete DMEM medium for 96 h. Cell number was determined using the cell proliferation kit I (MTT, Sigma-Aldrich, 11465007001). The concentration of gemcitabine required to inhibit cell proliferation by 50% (IC_50_) was calculated.

For expression analyses, PANC-1 cells were transfected with siRNAs in 12 well plates. 7 days after transfection, cells were exposed to 50 µg/ml gemcitabine or to medium only for 6 h. Cells were washed and maintained in complete DMEM medium for 48 h and total RNA was extracted.

### Generation of M0 and TAM macrophages from THP-1 cells and medium collection

THP-1 cells were treated with 100 ng/ml phorbol 12-myristate 13-acetate (PMA, Sigma-Aldrich, P8139) for 24 h in 6 well plates (1.5 × 10^6^ cells/well) or 6-well inserts (0.4 µm pore size, Falcon, 353090, 6.5 × 10^5^ cells/insert). Activated THP-1 cells were washed and maintained in complete RPMI-1640 medium for another 24 h. Cells were considered M0 macrophages at this stage. Medium was changed again and M0 conditioned medium was collected after 24 h. PANC-1 or Calu-3 cells were transfected, incubated with M0 or control conditioned medium and cell number was determined (MTT kit, Sigma-Aldrich, 11465007001).

For the generation of specific-KD tumor-associated-macrophages (TAM), M0 macrophages were activated in 6-well inserts and then co-cultured with transfected PANC-1 or Calu-3 cells for 72 h. Then, macrophages were isolated, medium was changed, and TAM conditioned medium was collected after 24 h incubation. Total RNA from tumor-associated-macrophages was extracted. PANC-1 or Calu-3 cells were incubated with associated TAM, M0, or control conditioned medium and cell number was determined (MTT kit, Sigma-Aldrich, 11465007001).

Collected media were centrifuged at 1200 rpm for 5 min to remove cell debris, filtered using 0.2 μm syringe filters (Sarstedt, 83.1826.001), and stored at -80 °C. For experimental procedures, all conditioned media were diluted 1:1 with fresh media.

### Statistics

Statistical analyses and graphs were performed using R software with ggplot2 and ggpubr packages. Once normality and homoscedasticity of the variances verified, multiple comparisons between control and different siRNAs were analyzed using ANOVA with Tukey’s post-hoc test. Data were visualized using box plots, the central mark indicates the median and edges indicate 25th and 75th percentiles. Whiskers extend to the largest or smallest point comprised within 1.5 × of the interquartile range from both edges.

## Supplementary Information

Supplementary Material 1.

## Data Availability

The fetal human pancreas sequencing data are available in the LUDC repository (www.ludc.lu.se/resources/repository) with the following accession numbers upon reasonable request: LUDC2021.10.12 (bulk RNA sequencing data), and LUDC2021.10.18 (single cell RNA sequencing data). PANC-1 sequencing data have been deposited (accession number: PRJNA1043644 (https://www.ncbi.nlm.nih.gov/bioproject/)).
