# Supplementary material for: Homeobox protein B6 and homeobox protein B8 control immune-cancer cell interactions in pancreatic cancer
Source: Mol Biomed. 2025 Jul 7;6:48. doi: 10.1186/s43556-025-00292-5 (PMC12229978; doi:10.1186/s43556-025-00292-5)
Supplement: Supplementary file 1 — Supplementary Material 1. [file 43556_2025_292_MOESM1_ESM.zip › Supplementary_Files.docx]

***Title Page***

**Homeobox protein B6 and homeobox protein B8 control immune-cancer cell interactions in pancreatic cancer.**

Ludivine Bertonnier-Brouty ^1,2^, Sara Bsharat ^1,2^, Kavya Achanta ^1,2^, Jonas Andersson ^2^, Thanya Pranomphon ^1,2^, Tania Singh ^1,2^, Tuomas Kaprio ^3,4,5^, Jaana Hagström^3,4,5,6^, Caj Haglund ^3,4,5^, Hanna Seppänen^3,4,5^, Rashmi B Prasad ^2, 7*^ and Isabella Artner ^1,2*^

^1^Lund Stem Cell Center, Lund University, Lund, Sweden

^2^Lund University Diabetes Center, Lund University, Malmö, Sweden

^3^Department of Surgery, Helsinki University Hospital, Helsinki, Finland

^4^Translational Cancer Medicine Research Program, Faculty of Medicine, University of Helsinki, Helsinki, Finland

^5^iCAN, Digital Cancer Precision Medicine, University of Helsinki and HUS Helsinki University Hospital

^6^Department of Oral Pathology and Radiology, University of Turku, Turku, Finland

^7^ Institute of Molecular Medicine, Finland (FIMM), Helsinki University, Helsinki, Finland.

*Correspondence.* ***** Isabella Artner [Isabella.artner@med.lu.se](mailto:Isabella.artner@med.lu.se) +46462223829

***** Rashmi B Prasad [Rashmi.prasad@med.lu.se](mailto:Rashmi.prasad@med.lu.se) +46704640275

**Supplementary Material**

**Supplementary Figures**

**Supplementary Figure S1.** Disease specific survival analyses in PDAC. Kaplan–Meier survival curves for PDACs according to HOXB6 (a) or HOXB8 (b) protein expression.

**Supplementary Figur**e **S2**. Transfection efficiency determined by RT-qPCR and western blot analyses. HOXB6 knockdown efficiency 48h and 7 days after transfection in PANC-1 (a), 7 days after transfection in AsPC1 (b) and western blot confirmation (c).

**Supplementary Figure S3**. Cell viability, apoptotic and stemness capacities are impaired in siHOXB6 and siHOXB8 transfected cells both in PANC-1 and AsPC1.

**Supplementary Figure S4.** Experimental set-up to quantify cell viability.

**Supplementary Figure S5.** Immune association with HOXB6 expression.

**Supplementary Figure S6.** Immune association with HOXB8 expression.

**Supplementary Figure S7.** Transfection efficiency determined by RT-qPCR analyses and Calu-3 sensitivity to TAM.

**Supplementary Tables**

**Supplementary Table S1.** Identification of genes controlling human pancreas development and subtype-specific pancreatic cancer**,** subtype-specific genes and the associated literature. *Supplementary excel file.*

**Supplementary Table S2.** Overall survival analyses.

**Supplementary Table S3.** HOXB6 and HOXB8 co-expression analyses in embryo, normal adult tissues and PDAC tissues using Spearman's rank correlation method. *Supplementary excel file.*

**Supplementary Table S4.** Pathways identified by Reactome using significant co-expressed genes with both HOXB6 and/or HOXB8 in PDAC, embryo and adult normal tissues. *Supplementary excel file.*

**Supplementary Table S5.** Deregulated genes between siHOXB6, siHOXB8 or siHOXB6B8 and control transcriptomes and deregulated genes with direct HOXB6 or HOXB8 binding sites. *Supplementary excel file.*

**Supplementary Table S6.** Primer sequences for qPCR.


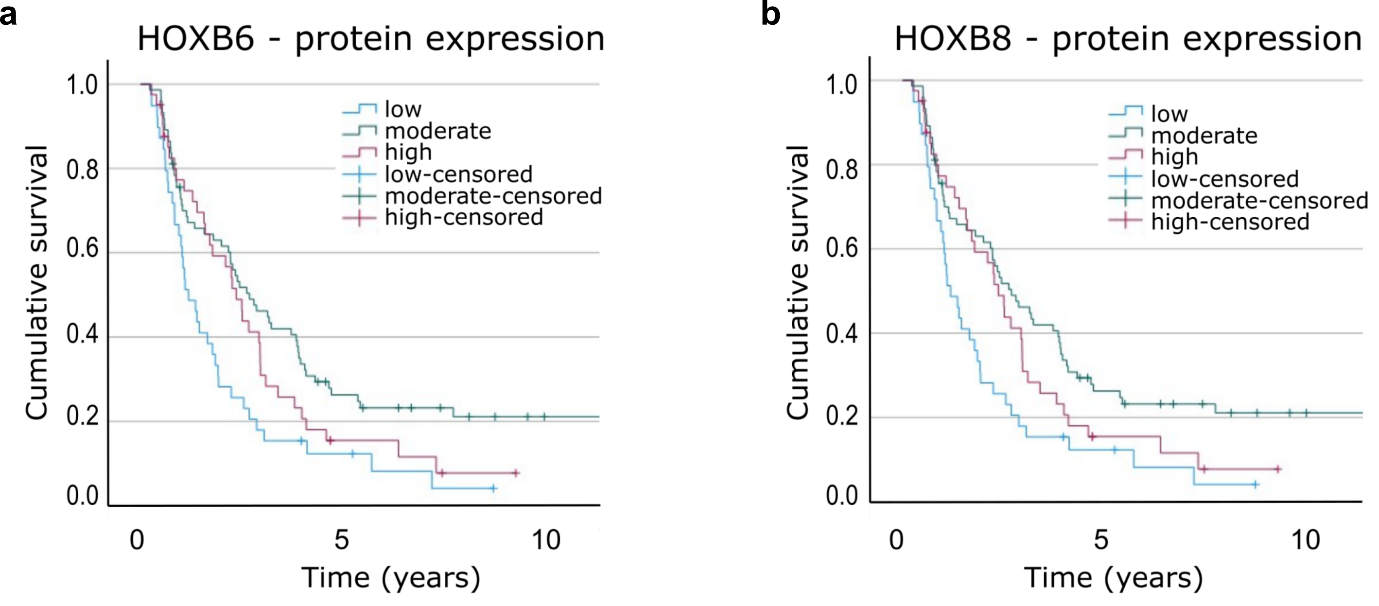


**Supplementary Figure S1.** Disease specific survival analyses in PDAC. Kaplan–Meier survival curves for PDACs according to HOXB6 (a) or HOXB8 (b) protein expression. Kaplan–Meier analysis revealed that low HOXB6 expression associated to poorer OS compared to moderate expression (p=0.005) or high expression (p=0.034). No difference is seen between moderate and high HOXB6 expression. 5-year OS for patients with a low HOXB6 expression was 12.3% (95% CI 1.8-22.19%) compared to moderate 26.3% (95% CI 16.1-36.5%) and high expression 15.5% (95% CI 4.1-26.9%). Low HOXB8 expression is associated with poorer OS compared to moderate expression (p=0.007) or high expression (p=0.007). No difference in OS is seen between moderate and high HOXB8 expression. 5-year OS for patients with a low HOXB8 expression was 9.7% (95% CI 2.8-16.6%) compared to moderate 21.4% (95% CI 12.0-30.8%) and high expression 24.0% (95% CI 12.7-36.7%).

**
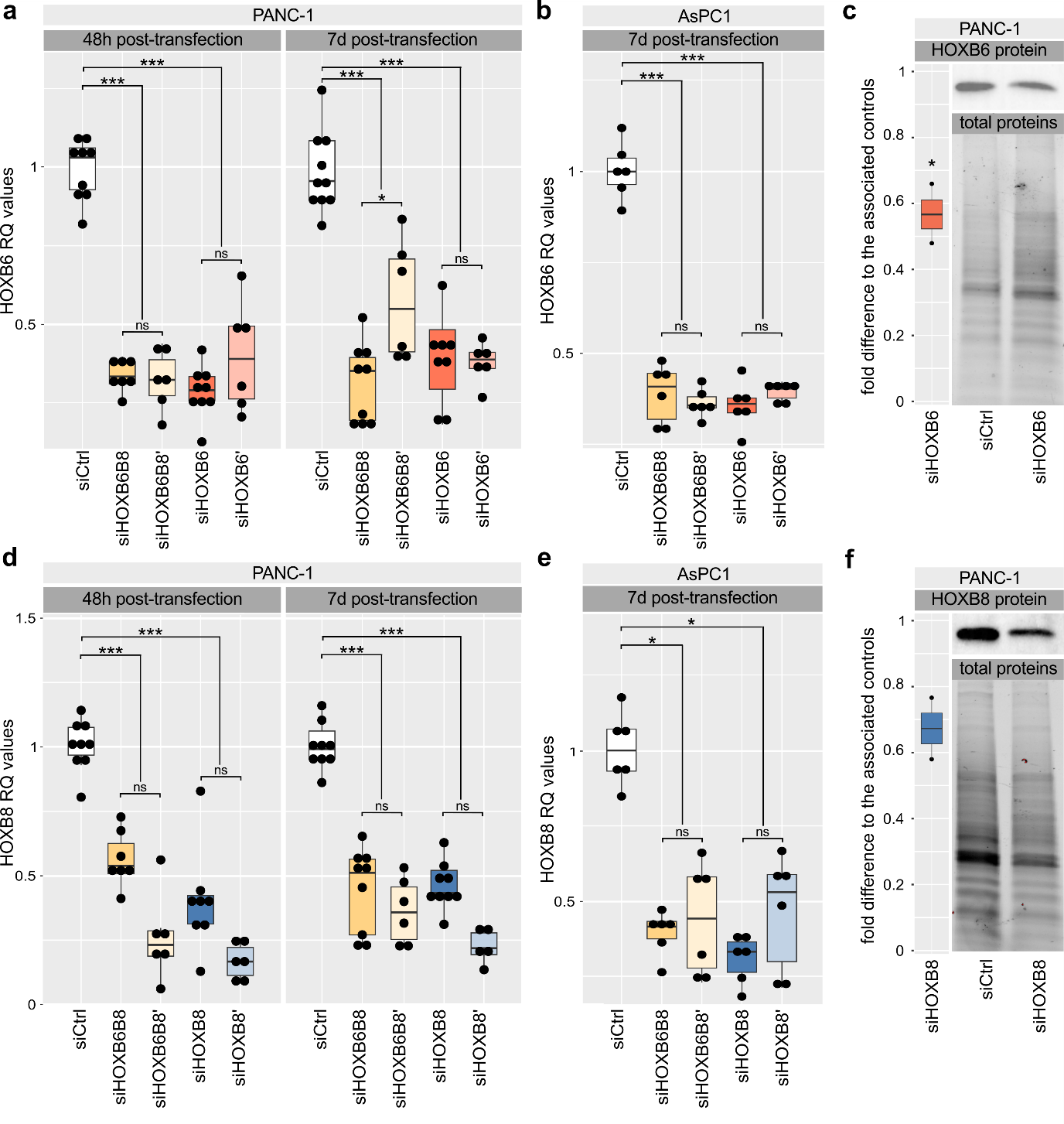
**

**Supplementary Figure S2.** Transfection efficiency determined by RT-qPCR and western blot analyses. HOXB6 knockdown efficiency 48h and 7 days after transfection in PANC-1 (a), 7 days after transfection in AsPC1 (b) and western blot confirmation (c). HOXB8 knockdown efficiency 48h and 7 days after transfection in PANC-1 (c), 7 days after transfection in AsPC1 (d) and western blot confirmation (f). (a, b, d, e) Expression values were calculated applying the −2∆∆CT algorithm. Estimated relative quantities were normalized for the expression value of genes S18 and TBP and calibrated to the negative control samples. Measurements were derived from three independent experiments. (c, f) Band density for target proteins was normalized against total protein load and fold change to the associated control were calculated. Tukey’s post-hoc test significances calculated on the ∆CT values and fold differences are indicated by stars compared to the control when significant. * p < 0.05, *** p < 0.001.


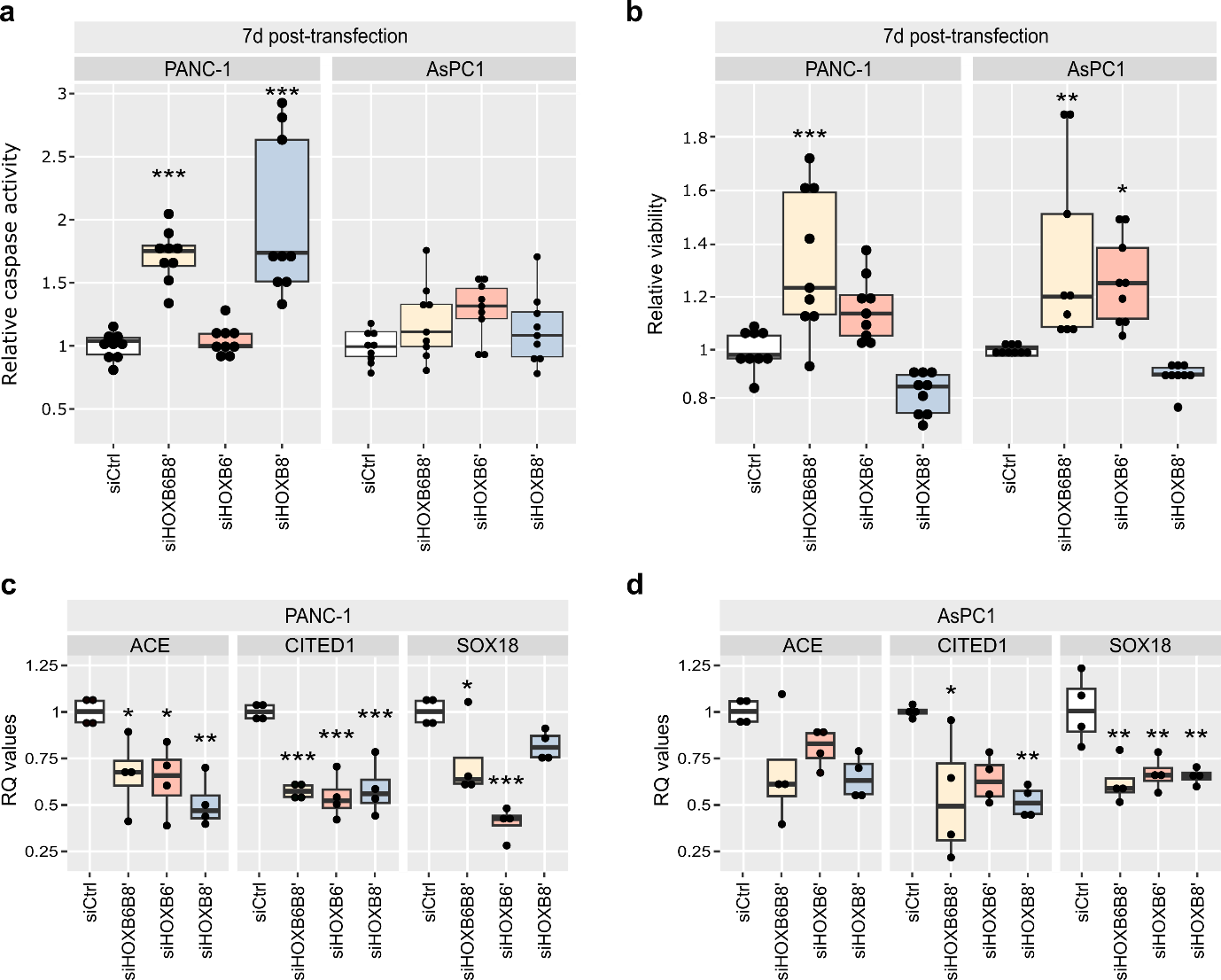


**Supplementary Figure S3** Cell viability, apoptotic and stemness capacities are impaired in siHOXB6 and siHOXB8 transfected cells both in PANC-1 and AsPC1. (a) Relative caspase activity 7 days after transfection in PANC-1 and AsPC1 using the second set of siRNAs. Results are shown as caspase-3/7 activity compared to negative control (n=9). (f) Cell viability 7 days post transfection in PANC-1 and AsPC1 using the second set of siRNAs. Results are shown as viability/cytotoxicity ratio compared to negative control (n=9). (c) 7 days after transfection of PANC-1 and AsPC1 cells, quantification of the expression of ACE, CITED1 and SOX18 cancer stem cell markers. Tukey’s post-hoc test significances are indicated by stars compared to the control when significant. * p < 0.05, ** p < 0.01, *** p < 0.001.


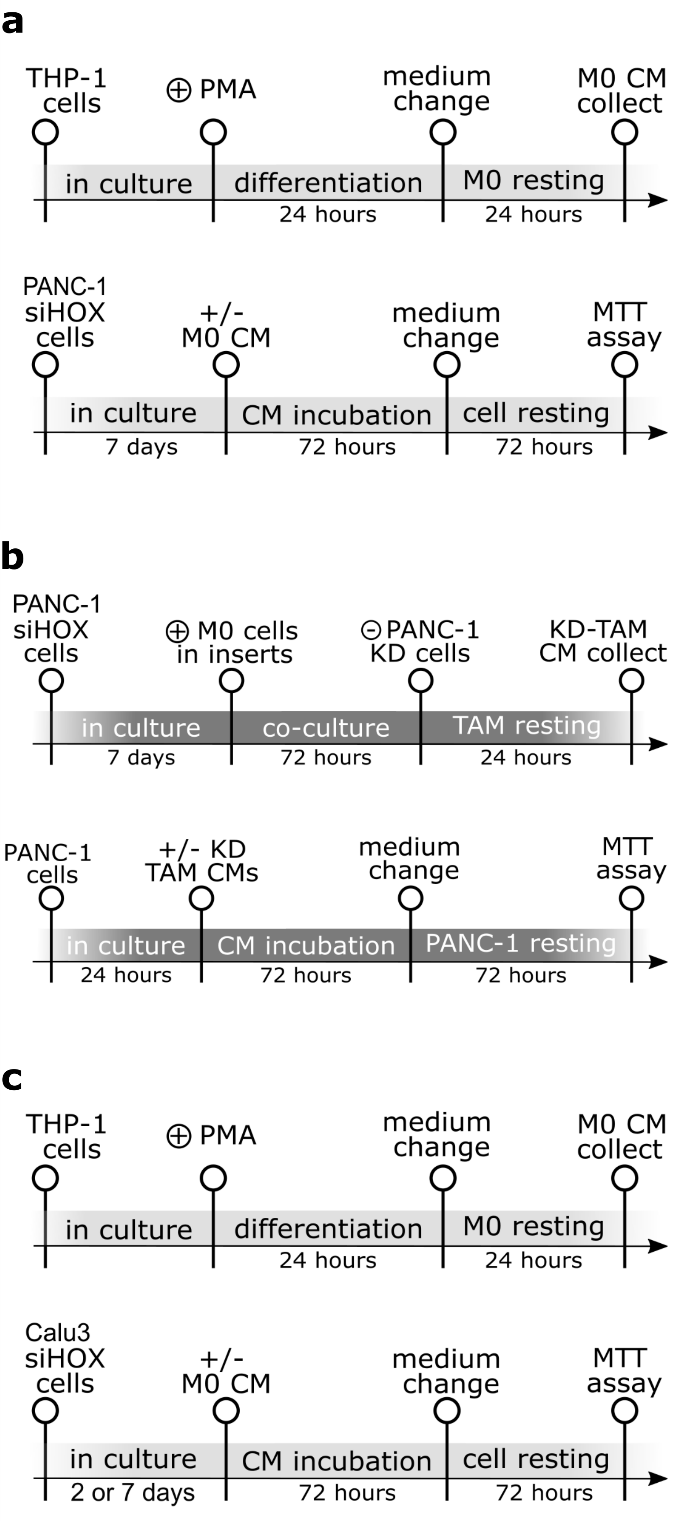


**Supplementary Figure S4. Experimental set-up to quantify** **cell viability**. (a) Preparation of naïve macrophages (M0) conditioned medium (CM) and viability test on PANC-1 siHOX cells. (b) Preparation of CM from siHOX-specific tumor-associated macrophages (TAMs) and viability test on PANC-1 cells. (c) Preparation of naïve macrophages (M0) conditioned medium (CM) and viability test on Calu3 siHOX cells.

**
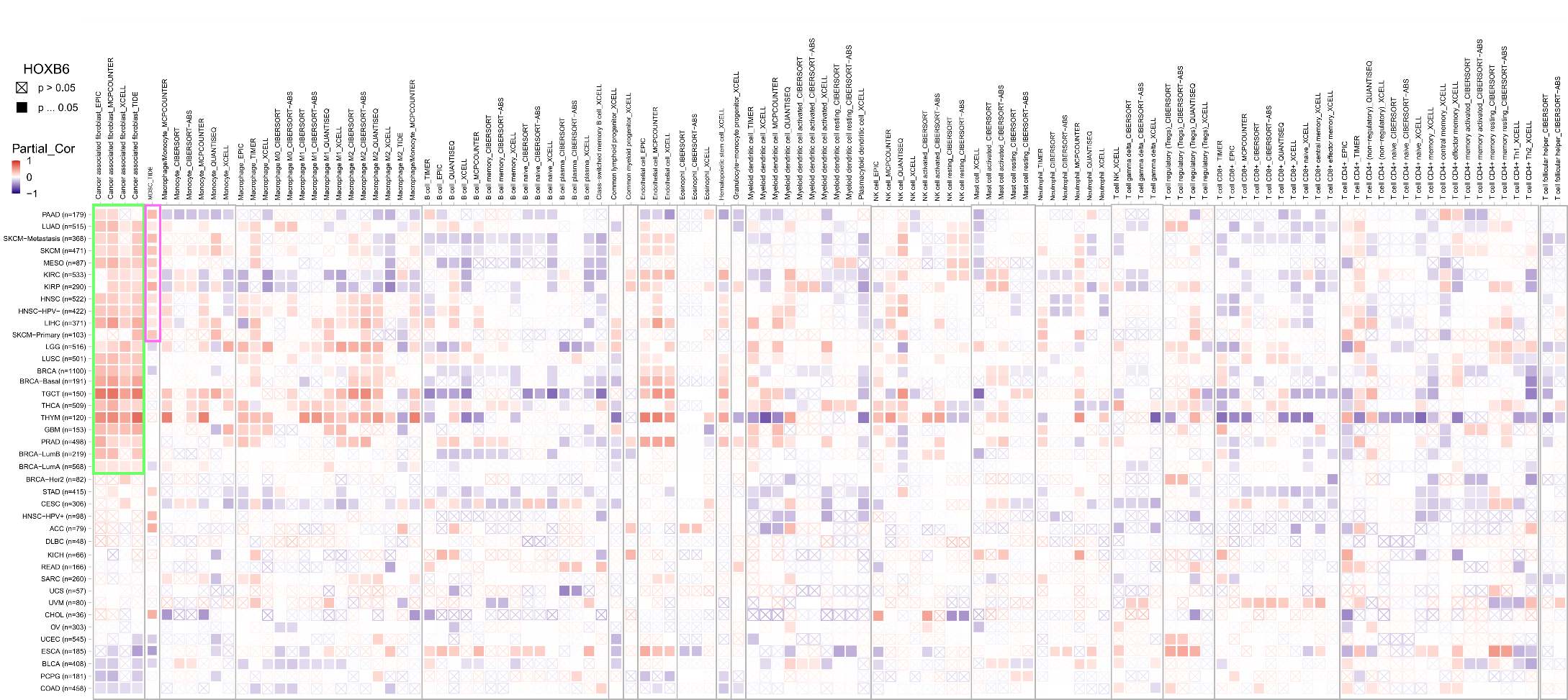
**

**Supplementary Figure S5. Immune association with HOXB6 expression.** TIMER2.0 analyses of HOXB6 expression correlating with immune infiltration in TCGA samples currently included in this analysis module [36-38]. Colored squares show significant results. Green rectangle shows cancer types where HOXB6 correlates with cancer associated fibroblasts, in pink, correlation with myeloid-derived suppressor cells (MDSC). Cancer abbreviations: ACC, Adrenocortical carcinoma; BLCA, Bladder Urothelial Carcinoma ; BRCA, Breast invasive carcinoma ; CESC, Cervical squamous cell carcinoma and endocervical adenocarcinoma ; CHOL, Cholangiocarcinoma ; COAD, Colon adenocarcinoma ; DLBC, Lymphoid Neoplasm Diffuse Large B-cell Lymphoma ; ESCA, Esophageal carcinoma ; GBM, Glioblastoma multiforme ; HNSC, Head and Neck squamous cell carcinoma ; KICH, Kidney Chromophobe; KIRC, Kidney renal clear cell carcinoma; KIRP, Kidney renal papillary cell carcinoma; LGG, Brain Lower Grade Glioma; LIHC, Liver hepatocellular carcinoma; LUAD, Lung adenocarcinoma; LUSC, Lung squamous cell carcinoma; MESO, Mesothelioma; OV, Ovarian serous cystadenocarcinoma; PAAD, Pancreatic adenocarcinoma; PCPG, Pheochromocytoma and Paraganglioma; PRAD, Prostate adenocarcinoma; READ, Rectum adenocarcinoma; SARC, Sarcoma; STAD, Stomach adenocarcinoma; SKCM, Skin Cutaneous Melanoma; TGCT, Testicular Germ Cell Tumors; THCA, Thyroid carcinoma ; THYM, Thymoma; UCEC, Uterine Corpus Endometrial Carcinoma; UCS, Uterine Carcinosarcoma; UVM, Uveal Melanoma.


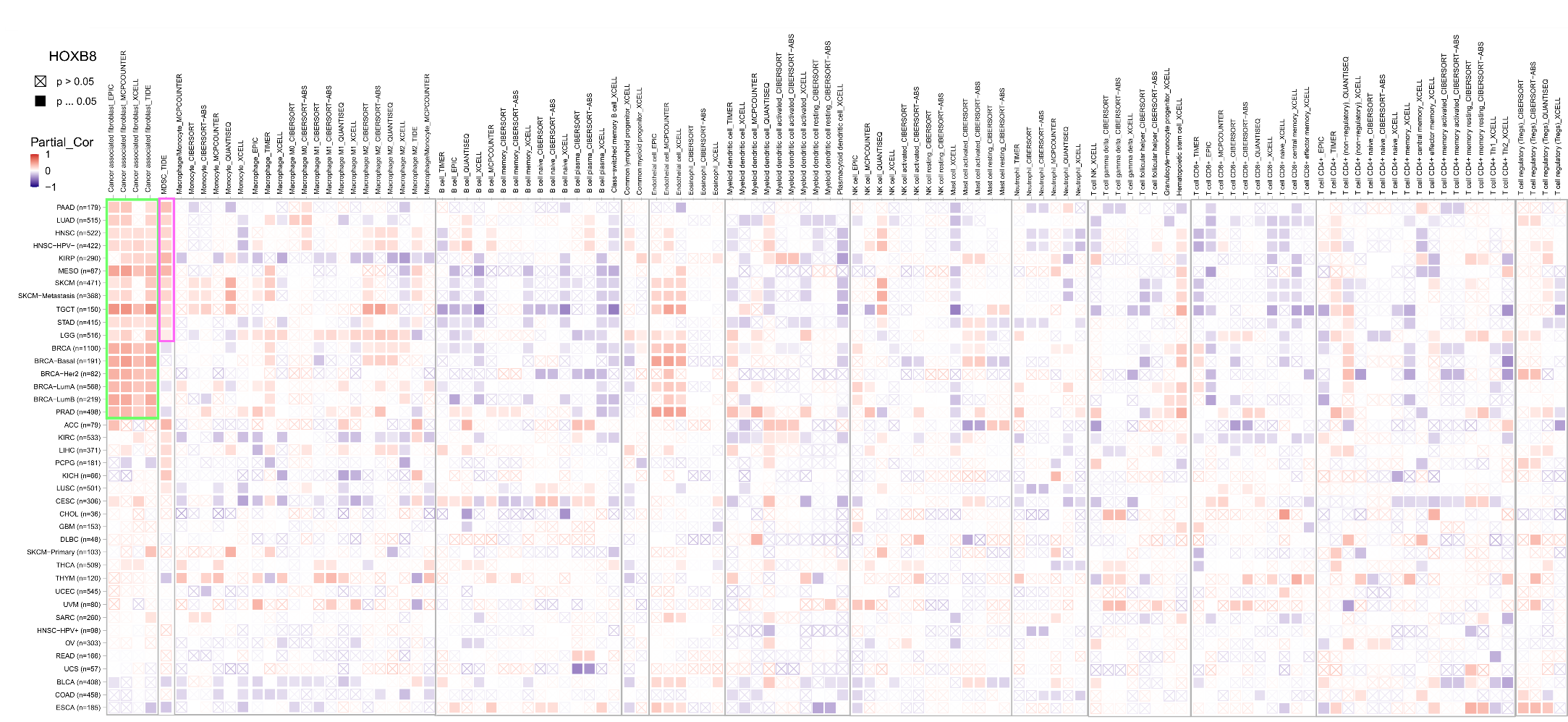


**Supplementary Figure S6. Immune association with HOXB8 expression.** TIMER2.0 analyses of HOXB8 expression correlating with immune infiltration in TCGA samples currently included in this analysis module [36-38]. Colored squares show significant results. Green rectangle shows cancer types where HOXB8 correlates with cancer associated fibroblasts, in pink, correlation with myeloid-derived suppressor cells (MDSC). Cancer abbreviations: ACC, Adrenocortical carcinoma; BLCA, Bladder Urothelial Carcinoma ; BRCA, Breast invasive carcinoma ; CESC, Cervical squamous cell carcinoma and endocervical adenocarcinoma ; CHOL, Cholangiocarcinoma ; COAD, Colon adenocarcinoma ; DLBC, Lymphoid Neoplasm Diffuse Large B-cell Lymphoma ; ESCA, Esophageal carcinoma ; GBM, Glioblastoma multiforme ; HNSC, Head and Neck squamous cell carcinoma ; KICH, Kidney Chromophobe; KIRC, Kidney renal clear cell carcinoma; KIRP, Kidney renal papillary cell carcinoma; LGG, Brain Lower Grade Glioma; LIHC, Liver hepatocellular carcinoma; LUAD, Lung adenocarcinoma; LUSC, Lung squamous cell carcinoma; MESO, Mesothelioma; OV, Ovarian serous cystadenocarcinoma; PAAD, Pancreatic adenocarcinoma; PCPG, Pheochromocytoma and Paraganglioma; PRAD, Prostate adenocarcinoma; READ, Rectum adenocarcinoma; SARC, Sarcoma; STAD, Stomach adenocarcinoma; SKCM, Skin Cutaneous Melanoma; TGCT, Testicular Germ Cell Tumors; THCA, Thyroid carcinoma ; THYM, Thymoma; UCEC, Uterine Corpus Endometrial Carcinoma; UCS, Uterine Carcinosarcoma; UVM, Uveal Melanoma.


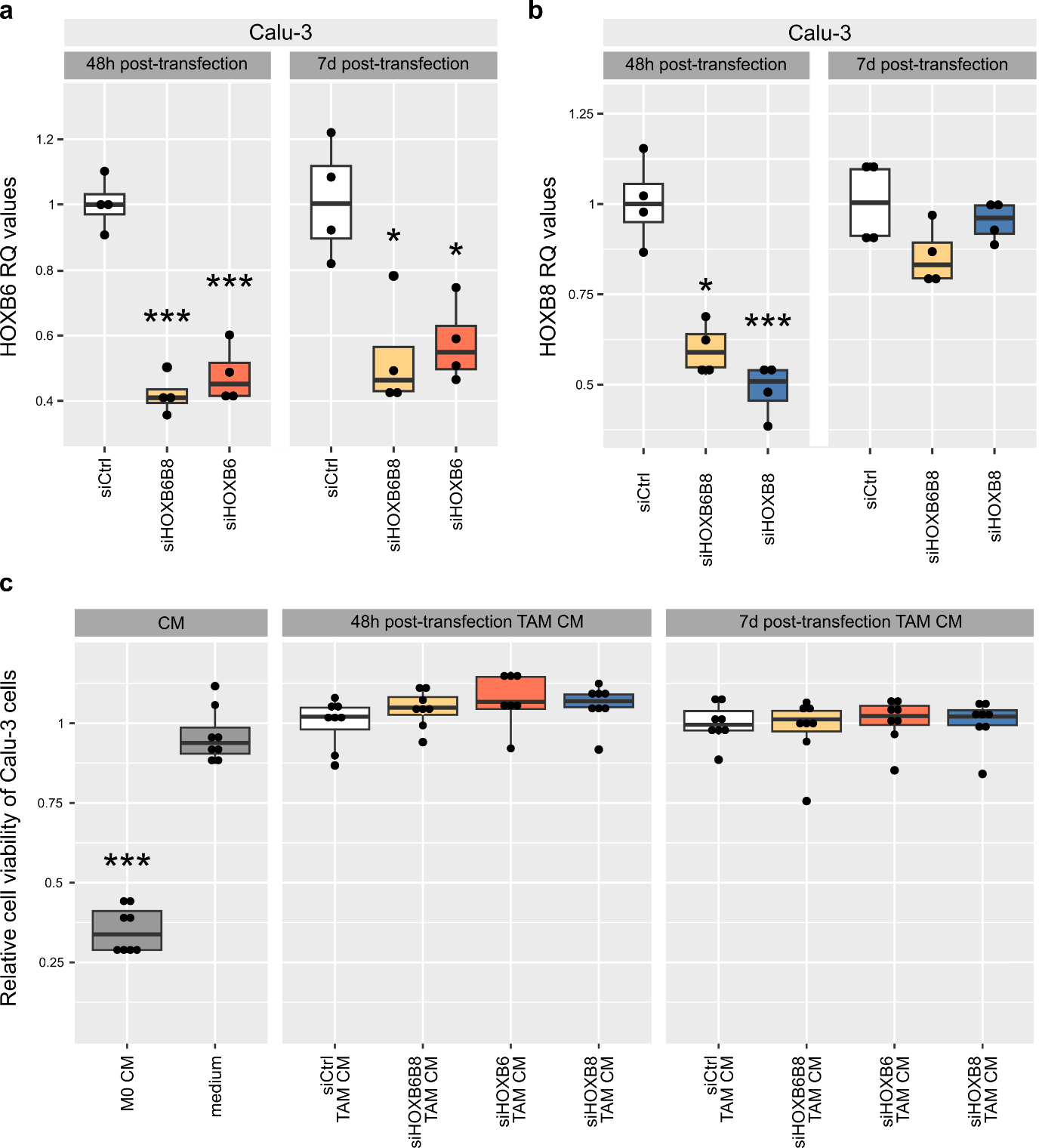


**Supplementary Figure S7.** **Transfection efficiency determined by RT-qPCR analyses and Calu-3 sensitivity to TAM.**  HOXB6 (a) and HOXB8 (b) knockdown efficiency 48h and 7 days post transfection in Calu-3 cells. Expression values were calculated applying the −2∆∆CT algorithm. Estimated relative quantities were normalized for the expression value of the endogenous genes S18 and TBP and calibrated to the negative control samples. Tukey’s post-hoc test significances calculated on the ∆CT values are indicated by stars compared to the control when significant. (c) MTT assay quantifying the relative cell viability of Calu-3 cells exposed to conditioned mediums from siHOX-specific TAMs, M0 or control culture medium compared to siCtrl TAM values. * p < 0.05, *** p < 0.001.

|  | HOXB6 | | | | HOXB8 | | | |
| --- | --- | --- | --- | --- | --- | --- | --- | --- |
|  | low | moderate | high | p-value | low | moderate | high | p-value |
|  | **18+21** | **74** | **41** |  | **17+14** | **76** | **47** |  |
| **Age** |  |  |  |  |  |  |  |  |
| <65 | 11 (28.2) | 21 (28.4) | 11 (26.8) | 0.98 | 9 (29.0) | 24 (31.6) | 10 (21.3) | 0.46 |
| ≥65 | 28 (71.8) | 53 (71.6) | 30 (73.2) |  | 22 (71.0) | 52 (68.4) | 37 (78.7) |  |
| **Sex** |  |  |  |  |  |  |  |  |
| Female | 17 (43.6) | 32 (43.2) | 20 (48.8) | 0.83 | 16 (51.6) | 30 (39.5) | 23 (48.9) | 0.41 |
| Male | 22 (56.4) | 42 (56.8) | 21 (5.12) |  | 15 (48.4) | 46 (60.3) | 24 (51.1) |  |
| **Stage (WHO)** |  |  |  |  |  |  |  |  |
| IA | 2 (5.1) | 3 (4.1) | 5 (12.2) | 0.83 | 2 (6.5) | 5 (6.6) | 3 (6.4) | 0.21 |
| IB | 4 (10.3 | 10 (13.5) | 3 (7.3) |  | 1 (3.2) | 13 (17.1) | 3 (6.4) |  |
| IIA | 5 (12.8) | 9 (12.2) | 6 (14.6) |  | 4 (12.9) | 8 (10.5) | 8 (17.0) |  |
| IIB | 27 (69.2) | 51 (68.9) | 26 (63.4) |  | 22 (71.0) | 50 (65.8) | 32 (68.1) |  |
| III | 1 (2.6) | 1 (1.4) | 1 (2.4) |  | 2 (6.5) | 0 (0.0) | 1 (2.1) |  |
| **pT** |  |  |  |  |  |  |  |  |
| 1 | 3 (7.7) | 3 (4.1) | 5 (12.2) | 0.52 | 3 (9.7) | 5 (6.6) | 3 (6.4) | **0.025** |
| 2 | 8 (20.5) | 24 (32.4) | 8 (19.5) |  | 3 (9.7) | 28 (36.8) | 9 (19.1) |  |
| 3 | 27 (69.2) | 46 (62.2) | 27 (64.9) |  | 23 (74.2) | 43 (56.6) | 34 (72.3) |  |
| 4 | 1 (2.6) | 1 (1.4) | 1 (2.4) |  | 2 (6.5) | 0 (0.0) | 1 (2.1) |  |
| **pN** |  |  |  |  |  |  |  |  |
| negative | 11 (28.2) | 23 (31.1) | 14 (34.1) | 0.85 | 7 (22.6) | 28 (36.8) | 13 (27.7) | 0.29 |
| positive | 28 (71.8) | 51 (68.9) | 27 (65.9) |  | 24 (77.4) | 48 (63.2) | 34 (72.3) |  |
| **Grade (WHO)** |  |  |  |  |  |  |  |  |
| 1 | 5 (12.8) | 7 (9.5) | 6 (15.0) | 0.32 | 3 (9.7) | 11 (14.5) | 4 (8.7) | 0.20 |
| 2 | 24 (61.5) | 56 (75.7) | 30 (75.0) |  | 19 (61.3) | 54 (71.1) | 37 (80.4) |  |
| 3 | 10 (25.6) | 11 (14.9) | 4 (10.0) |  | 9 (29.0) | 11 (14.5) | 5 (10.9) |  |
| **LNR** |  |  |  |  |  |  |  |  |
| <20% | 29 (74.4) | 56 (75.7) | 33 (82.5) | 0.63 | 22 (71.0) | 56 (73.7) | 40 (87.90) | 0.16 |
| >20% | 10 (25.6) | 18 (24.3) | 7 (17.5) |  | 9 (29.0) | 20 (26.3) | 6 (13.0) |  |
| **Perineural invasion** |  |  |  |  |  |  |  |  |
| No | 10 829.4) | 13 (20.3) | 7 (21.2) | 0.57 | 5 (19.2) | 14 (21.5) | 11 (27.5) | 0.69 |
| Yes | 24 (70.6) | 51 (79.7) | 26 (78.8) |  | 21 (80.8) | 51 (78.5) | 29 (72.5) |  |
| **Perivascular invasion** |  |  |  |  |  |  |  |  |
| No | 25 (71.4) | 39 (65.0) | 10 (61.3) | 0.87 | 19 (70.4) | 37 (61.7) | 27 (69.2) | 0.64 |
| Yes | 10 (28.6) | 21 (35.0) | 12 (38.7) |  | 8 (29.6) | 23 (38.3) | 12 (30.8) |  |

**Supplementary Table S2.** Overall survival analyses

| Primers | Forward | Reverse |
| --- | --- | --- |
| ACE | ACGTGAGGATACAGCAAGGC | AGAGTTCCTGCATGGTCTGG |
| CD163 | GAGTCAGCTCTTTGGGATTGC | CCAAATTGGATCCATCTGAGC |
| CD206 | GCGTGGCTGCAGATGGAAACATCTA | TGTACCTCACCCTCCACTTATCAGTCCA |
| CD209 | GCAGTCTTCCAGAAGTAACCGC | GCTCTCCTCTGTTCCAATACTGC |
| CD80 | GGGAAATGTCGCCTCTCTGA | GTGGATTTAGTTTCACAGCTTG |
| CD86 | CCATCAGCTTGTCTGTTTCATTCC | GCTGTAATCCAAGGAATGTGGTC |
| CDKN1A | AGGTGGACCTGGAGACTCTCAG | TCCTCTTGGAGAAGATCAGCCG |
| CDKN2A | GGAAGCAAATGTAGGGGTAA | CCAGCGTGTCCAGGAAG |
| CITED1 | AGGATGCCAACCAAGAGATG | GTTTAGTGGGAGGGGTGGTT |
| GAPDH | TGGTATCGTGGAAGGACTCATGAC | TGCCAGTGAGCTTCCCGTTCAGC |
| GLB1 | GTTATAACAGTGCAGGTTGAAAATGAA | CCCAGATGGTGGCGAAAG |
| HOXB6 | AGCAGCCCCCGTTCCA | AAAGGAGGAACTGTTGCACGAAT |
| HOXB8 | TAAGCGGCGAATCGAGGTAT | TGTTTCTCCAGCTCCTCCTG |
| RPL37A | ATTGAAATCAGCCAGCACGC | ATTGAAATCAGCCAGCACGC |
| S18 | AGCTTGTTGTCCAGACCATT | TGAGGAAAGCAGACATTGAC |
| SOX18 | TTCCATGTCACAGCCCCCTAG | GACACGTGGGAACTCCAG |
| TBP | GAACATCATGGATCAGAACAACA | ATAGGGATTCCGGGAGTCAT |
| TLR2 | CTTCACTCAGGAGCAGCAAGCA | ACACCAGTGCTGTCCTGTGACA |
| TLR4 | CCCTGAGGCATTTAGGCAGCTA | AGGTAGAGAGGTGGCTTAGGCT |

**Supplementary Table S6.** Primer sequences for qPCR.
